# The Genus *Terminalia* (Combretaceae): An Ethnopharmacological, Phytochemical and Pharmacological Review

**DOI:** 10.1007/s13659-019-00222-3

**Published:** 2019-11-06

**Authors:** Xiao-Rui Zhang, Joseph Sakah Kaunda, Hong-Tao Zhu, Dong Wang, Chong-Ren Yang, Ying-Jun Zhang

**Affiliations:** 1 State Key Laboratory of Phytochemistry and Plant Resources in West China. Kunming Institute of Botany, Chinese Academy of Sciences, Kunming, 650204 People’s Republic of China; 2 University of Chinese Academy of Sciences, Beijing, 100049 People’s Republic of China; 3 Yunnan Key Laboratory of Natural Medicinal Chemistry, Kunming Institute of Botany, Chinese Academy of Sciences, Kunming, 650201 People’s Republic of China

**Keywords:** *Terminalia*, Combretaceae, Ethnomedicine, Traditional uses, Phytochemistry, Hydrolyzable tannins, Pharmacology

## Abstract

*Terminalia* Linn, a genus of mostly medium or large trees in the family Combretaceae with about 250 species in the world, is distributed mainly in southern Asia, Himalayas, Madagascar, Australia, and the tropical and subtropical regions of Africa. Many species are used widely in many traditional medicinal systems, e.g., traditional Chinese medicine, Tibetan medicine, and Indian Ayurvedic medicine practices. So far, about 39 species have been phytochemically studied, which led to the identification of 368 compounds, including terpenoids, tannins, flavonoids, phenylpropanoids, simple phenolics and so on. Some of the isolates showed various bioactivities, in vitro or in vivo, such as antitumor, anti HIV-1, antifungal, antimicrobial, antimalarial, antioxidant, diarrhea and analgesic. This review covers research articles from 1934 to 2018, retrieved from SciFinder, Wikipedia, Google Scholar, Chinese Knowledge Network and Baidu Scholar by using “*Terminalia*” as the search term (“all fields”) with no specific time frame setting for the search. Thirty-nine important medicinal and edible *Terminalia* species were selected and summarized on their geographical distribution, traditional uses, phytochemistry and related pharmacological activities.

## Introduction

*Terminalia* Linn, comprising about 250 species in the world mostly as medium or large trees, is the second largest genus in the family Combretaceae. The name “*Terminalia*” is derived from Latin word “terminus”, which means the leaves are located at the tip of the branch. The bark of *Terminalia* plants usually has cracks and branches tucked into layers. Most of the *Terminalia* plants’ leaves are large, leathery with solitary or clustered small green white flowers. Their fruits are yellow, dark red or black; drupe, usually angular or winged. Some fruits are edible, highly nutritious and possess medicinal values.

*Terminalia* species are widely distributed in the southern Asia, Himalayas, Madagascar, Australia, and the tropical and subtropical regions of Africa. *Terminalia* plants in southern Asia have been intensively studied phytochemically due to their wide usage in Asian (India, Tibetan, and Chinese) traditional medicine systems [1]. For example, the fruits of *Terminalia bellirica* and *Terminalia chebula*, together with *Phyllanthus emblica* (Euphorbiaceae) which form the herbal remedy, Triphala, in Tibetan medicine, have received much attention because of its extensive and remarkable effectiveness in the treatment of anticancer, antifungal, antimicrobial, antimalarial, antioxidant.

So far, 39 *Terminalia* species have been investigated for their phytochemical constituents, which resulted in the identification of terpenes, tannins, flavonoids, lignans and simple phenols, amongst others. Pharmacological studies suggest that they have exhibited activity on liver and kidney protection, antibacterial, antiinflammatory, anticancer, and have displayed a positive effect on immune regulation, cardiovascular disease and diabetes, and acceleration of wound healing.

This paper features 39 important medicinal and edible *Terminalia* species and summarizes their traditional usage, geographical distribution, structures of isolated chemical constituents and pharmacological activities.

## Species’ Description, Distribution and Traditional Uses

So far, 50 *Terminalia* species have been documented, 39 of which have been reported to possess medicinal properties and/or being edible. Among them, eight species and four varieties including *T. argyrophylla*, *T. bellirica*, *T. catappa*, *T. chebula*, *T. franchetii*, *T. hainanensis*, *T. myriocarpa*, *T. intricate*, *T. chebula* var. *tomentella*, *T. franchetii* var. *membranifolia*, *T. franchetii* var. *glabra*, and *T. myriocarpa* var. *hirsuta* are distributed in China (Yunnan, southeast Tibet, Taiwan, Guangdong, south Guangxi and southwest Sichuan). Their distribution and traditional applications are shown in Table 1.

**Table 1:** Local names, distributions and traditional uses of *Terminalia* plants

No.	Plants	Local names	Distributions	Traditional uses
T1	*T. alata*	Unknown	Southern Vietnam [2, 3]	Anti-diarrhea, ulcer, diuretics, supplements [3]
T2	*T. amazonia*	White olive	Southern Costa Rica [4]	Wood
T3	*T. arborea*	Jaha Kling	Indonesia	Cardiovascular disease, myocardial infarction, atherosclerosis, diabetes, cancer, stroke, cataract, shoulder stiffness, cold allergy, hypertension, senile dementia, inflammation, gum disease (e.g. gingivitis, pneumonia), Alzheimer’s, skin conditions [5]
T4	*T. arjuna*	Arjuna, White Marudah, Koha	India, South Asia, Sri Lanka [6]	Cardiotonic, sores, bile infection, poison antidote [6] Coughs, dysentery, fractures, contusions, ulcers, hypertension ischaemic heart diseases [23]
T5	*T. argyrophylla*	Silver leaves Chebula, Xiao Chebula (Yunnan), Manna (Yunnan Dai language)	China (Yunnan) [7]	Autoimmune diseases [7]
T6	*T. australis*	Tanimbu, palo amarillo	Punta Lara, Argentina (Buenos Aires) [8]	Hemostasis
T7	*T. avicennioides*	kpayi, Kpace, baushe	Nigeria [9, 10]	Malaria, worms, gastric peptic ulcer [9], scorpion bites [10], tuberculosis, cough [90]
T8	*T. bellirica*	Beleric	China (southern Yunnan), Vietnam, Laos, Thailand, Cambodia, Myanmar, India (except West), Malaysia, Indonesia	Laxative, edible Edema, diarrhea, leprosy, bile congestion, indigestion, headache [11] Fever, diarrhea, cough, dysentery, skin diseases [12] Wine, palm sugar [23] Diarrhea [94]
T9	*T. bentzoe*	Unknown	Rodrigues [13]	Essential oil [13]
T10	*T. bialata*	Indian silver greywood	India, South Asia	Wood [14]
T11	*T. brachystemma*	Kalahari cluster leaf	Southern Africa	Shistosomiasis, gastrointestinal disorders [15]
T12	*T. brownii*	kuuku, muvuku (Kamba, Kenya), koloswa (northern region, Kenya), weba (Ethiopia), lbukoi (Samburu, Kenya), orbukoi (Maasai, Tanzania), and mbarao or mwalambe, in Kiswahili	Southern and central Africa	Diarrhea, stomach pain, gastric ulcer, colic, heartburn Genitourinary infection, urethral pain, endometritis, cystitis, leucorrhea, syphilis, gonorrhea, malaria, dysmenorrhea, nervousness, hysteria, epilepsy, athlete’s foot, indigestion, stomach pain, gastric ulcer, colitis, cough, vomiting, hepatitis, jaundice, cirrhosis, yellow fever [16]
T13	*T. bursarina*	Yellow wood	Australia, South Asia [17]	Unknown
T14	*T. calamansanai*	Phillipine almond, Anarep	Philippines, Southeast Asia	Lithontriptic [18], horticultural plant [102]
T15	*T. calcicola*	Unknown	Madagascar Rain Forest [19]	Unknown
T16	*T. catappa*	Indian almond, umbrella tree, tropical almond	China (Guangdong, Taiwan, SE Yunnan), Australia and SE Asia, Africa, South America Tropical Coast	Blood stasis, liver injury [20] Diarrhea, dysentery, biliary inflammation [23], dermatitis, hepatitis [106]
T17	*T. chebula*	Black Mytrobalan, Inknut, Chebulic Myrobalan	Nepal, northern India, Myanmar, Sri Lanka, Thailand, Bangladesh, China (Yunnan), Himalayan	Digestion appetizers, vomiting, infertility, asthma, sore throat, vomiting, urticaria, diarrhea, dysentery, bleeding, ulcers, gout, bladder disease [21]
T18	*T. chebula* var. *tomentella*	Weimaohezi (variant)	China (western Yunnan), Myanmar	Unknown
T19	*T. citrina*	Manahei, Yellow myrobalan	India, Bangladesh [22]	Dysmenorrhea, bleeding, heart disease, dysentery, constipation [22]
T20	*T. elliptica*	Indian laurel	SE Asia, India, Bangladesh, Laos, Myanmar, Nepal, Thailand, Cambodia, Vietnam	Wine, palm sugar Ulcers, fractures, bleeding, bronchitis, diarrhea [23]
T21	*T. franchetii*	Dianlanren	SW China [24]	Unknown
T22	*T. franchetii* var. *membranifolia*	Baoyedianlanren (variant)	China [western Guangxi (Longlin), central to SE Yunnan]	Unknown
T23	*T. franchetii* var. *glabra*	Guang yedianlanren (variant)	China (Sichuan and Yunnan Jinsha River Basin)	Unknown
T24	*T. ferdinandiana*	Gubinge, Bbillygoat plum, Kakadu plum, green plum, salty plum, murunga, mador	Australia [25]	Dietary supplements, skin care [25]
T25	*T. glaucescens*	Unknown	Nigeria [26]	Amenorrhea, vaginal infections, syphilis, sores, neurological disorders Anti-plasma, antiparasitic, antiviral, antimicrobial [26, 27]
T26	*T. hainanensis*	Ji zhenmu, Hainan lanren	China (Hainan)	Antioxidant [28]
T27	*T. intricate*	Cuozhilanren	China (NW Yunnan and SW Sichuan)	Unknown
T28	*T. ivorensis*	Idigbo, Black Afara, Shingle Wood, Brimstone Wood, Blackbark	Cameroon, West Africa, Ivory Coast, Liberia, Nigeria, Sierra Leone, Ghana	Rheumatism, gastroenteritis, psychotic analgesics [29] Syphilis, burns and bruises [30]
T29	*T. kaernbachii*	Okari Nut	Solomon Islands, Papua New Guinea	*α*-Glucosidase inhibitor activity [31]
T30	*T. kaiserana*	Unknown	Tanzania	Diarrhea, gonorrhea vomiting [44]
T31	*T. laxiflora*	Unknown	West Africa, Sudan Savannah	Malaria, cough [32] Fumigant, rheumatic pain, smoothen skin, body relaxation [33]
T32	*T. macroptera*	Bayankada	Tropical (West Africa)	Wound, hepatitis, malaria, fever, cough, diarrhea, tuberculosis, skin diseases [34]
T33	*T. mantaly*	Unknown	Africa, Madagascar	Dysentery
T34	*T. mollis*	Bush willow	Africa	Diarrhea, gonorrhea, malaria, AIDS adjuvant therapy [35]
T35	*T. muelleri*	Ketapang kencana	Indonesia, SE Asia, South Asia	Antibacterial [36], antioxidants [37]
T36	*T. myriocarpa*	Qianguolanren	China [Guangxi (Longjin), Yunnan (central to the south), and Tibet (Medog)], northern Vietnam, Thailand, Laos, northern Myanmar, Malaysia, NE India, Sikkim	Antioxidant, liver protection [38]
T37	*T. myriocarpa* var. *hirsuta*	Yingmaoqianguolanren (variant)	Yunnan, China; Thailand	Unknown
T38	*T. oblongata*	Rose wood, yellow wood	Central Queensland [39]	Unknown [39]
T39	*T. paniculata*	Vellamaruth	India	Cholera, mumps, menstrual disorders, cough, bronchitis, heart failure, hepatitis, diabetes, obesity [40]
T40	*T. parviflora*	Tropical almond, umbrella tree, Indian almond	Sri Lanka and India [41]	Diarrhea [41]
T41	*T. prunioides*	Hareri, Sterkbos, Purple pod Terminalia, Mwangati	Southern Africa	Postnatal abdominal pain
T42	*T. sambesiaca*	Unknown	Southern Africa	Cancer, gastric ulcer, appendicitis Bloody diarrhea [45]
T43	*T. schimperiana*	Idi odan	Africa, Sierra Leone, Guinea, Uganda, Ethiopia	Local burns, bronchitis, dysentery [42]
T44	*T. sericea*	Monakanakane, Mososo, Mogonono, Amangwe, Vaalboom, Mangwe, Silver clutter-leaf	Northern South Africa, Botswana (except central Kalahari), southern Mozambique, Tanzania, Namibia, Zimbabwe, Northern Democratic Republic of Congo, tropical Africa [43]	Diarrhea, sexually transmitted infections, rash, tuberculosis [43] Fever, high blood pressure [44]
T45	*T. spinosa*	Musosahwai, spiny cluster leaf, Kasansa	Southern Africa	Malaria, fever [46] Epilepsy, poisoning [47]
T46	*T. stenostachya*	Rosette leaf Terminalia	Southern Africa	Epilepsy, poisoning [47]
T47	*T. stuhlmannii*	Unknown	Acacia [48]	Unknown
T48	*T. superba*	Limba	Tropical Western Africa	Gastroenteritis, diabetes, female infertility, abdominal pain, bacteria/fungi/viral infections [49], diabetes remedies, anesthetic, hepatitis [50]
T49	*T. triflora*	Lanza, lanza amarilla, amarillo derío, paloamarillo	Tropical (South America) Northern and Northwest Argentina [149]	Making posts, furniture, weapons, fuel [149]
T50	*T. tropophylla*	Unknown	Madagascan [51]	Unknown

*SE* southeastern, *NE* northeastern, *SW* southwestern, *NW* northwestern

*Terminalia* species are broadly used in many aspects. Some are employed as drugs, while others can provide high quality wood, tannin or dyes. For example, fruits of *T. ferdinandiana*, a species largely distributed in Australia, are rich in vitamin C, and possess strong antioxidant activity [25]. *T. bellirica* and *T. chebula* are not only recorded in every version of Chinese pharmacopoeia, but are also the important and most commonly applied drugs in Han, Tibetan, Mongolian and many other folk medicinal systems in India, Burma, Thailand, Malaysia, Vietnam and other southeast asian countries. *T. catappa* is a commonly used medicinal plant for liver protection in China [20].

## Chemical Composition

Since 1930s, the chemical compositions of the genus *Terminalia* have been vastly studied. *T. arjuna*, *T. bellirica*, *T. catappa* and *T. chebula*, having been frequently used in the Ayurvedic, Chinese and Tibetan medicines, attracted scholars’ attention. To date, 368 compounds, largely terpenoids (**1–104**), tannins (**105–196**), flavonoids (**197–241**), lignans (**242–265**), phenols and glycosides (**268–318**) were reported from the genus (Tables 2, 3).

**Table 2:** Chemical constituents isolated from the genus *Terminalia* and the studied plant organs

No.	Compounds	Plants	Organs	References
Triterpenes (86)				
**1**	2α,3β,19α-Trihydroxyolean-12-en-20-oic acid 3-*O*-β-d-galactosyl-(1 → 3)-β-d-glucoside	T1	R	[3]
**2**	2α,3β,19α-Trihydroxyolean-12-en-28-oic acid methylester 3β-*O*-rutinoside	T1	R	[53]
**3**	2α,3β,19β,23-Tetrahydroxyolean-12-en-28-oic acid 3β-*O*-β-d-galactosyl-(1 → 3)-β-d-glucoside-28-*O*-β-d-glucoside	T1	R	[52]
**4**	3-Acetylmaslinic acid	T1	RB	[54]
**5**	Arjunic acid	T1 T4 T17 T25 T28 T32 T44	B SB, F F SB B B R	[55, 74] [60, 79, 124] [146] [130] [132] [145] [133]
**6**	Arjunoside I	T4	SB	[61]
**7**	Arjunoside II	T4	SB	[61]
**8**	Arjunoside III	T4	R	[62, 63]
**9**	Arjunoside IV	T4	R	[62, 63]
**10**	Arjunetin	T1 T4 T8, T16, T17, T20, T39	B B, L, S, R, F B, L, S, R, F	[55, 74] [23, 67] [23]
**11**	Oleanolic acid	T1 T9 T4, T16, T20 T8, T17 T39 T28 T36	H L B, L, S, R, F B, L, S, R L, S, R, F B B	[56] [97] [23] [23] [23] [132] [140]
**12**	Ursolic Acid	T4, T16, T20 T8, T17 T39	B, L, S, R, F L, S, R B, L, S, F	[23] [23] [23]
**13**	Maslinic acid	T1 T9 T17 T36	H L F B	[56] [97] [21, 116] [140]
**14**	2α,3α,24-Trihydroxyolean-11,13(18)-dien-28-oic acid	T33	SB	[158]
**15**	Terminoside A	T4	B	[58]
**16**	Arjungenin	T4 T25 T12 T8, T16, T20, T39 T17 T25 T28 T32 T33 T44	SB,L,R,F R B B, L, S, R, F B, L, S, R, F R, SB B B SB RB	[23, 60, 70, 74] [60] [99] [23] [23, 146] [69, 130] [132] [145] [158] [133, 152]
**17**	Hypatic acid	T25	R	[69]
**18**	Arjunglucoside I	T4 T17 T50 T32	B, R F R B	[70, 74, 78] [146] [72] [145]
**19**	Sericoside	T4 T25 T28 T44 T32 T50	B SB B R, L, SB B R	[71] [130] [76, 131] [43, 133, 149] [145] [72]
**20**	Crataegioside	T4 T17	B F	[75] [146]
**21**	23-*O*-neochebuloylarjungenin 28-*O*-β-d-glycosyl ester	T17	F	[146]
**22**	23-*O*-4′-*epi*-neochebuloylarjungenin	T17	F	[146]
**23**	23-*O*-galloylarjunic acid	T39 T32	B B	[144] [145]
		T17	F	[146]
**24**	Quercotriterpenoside I	T32	B	[145]
		T17	F	[146]
**25**	Sericic acid	T28 T32 T44	B B R	[132] [145] [150]
**26**	24-Deoxy-sericoside	T32	B	[138]
**27**	Arjunolic acid	T1 T4 T7 T9 T8 T16, T17, T20, T39 T34 T36	B, H B, H, L, S, R, F RB L B, L, S, R B, L, S, R, F L B	[55, 56, 74] [23, 77, 78, 91] [97] [23] [23] [23, 144] [35] [140]
**28**	Terminolic acid	T1 T17 T7, T16, T31 T25 T32	H F H H, Rl H, B	[56] [146] [128] [128] [128, 145]
**29**	Arjunglucoside II	T4 T17	B F	[70, 74] [146]
**30**	23-*O*-galloylarjunolic acid	T17	F	[146]
**31**	23-*O*-galloylarjunolic acid 28-*O*-β-d-glucosyl ester	T17	F	[146]
**32**	23-*O*-galloylterminolic acid 28-*O*-β-d-glucosyl ester	T17	F	[146]
**33**	Arjunolitin	T4	SB	[80]
**34**	Terminolitin	T4	F	[80]
**35**	Arjunglucoside III	T4	B	[74]
**36**	Methyl oleanate	T4	R, F	[80, 124]
**37**	Olean-3α,22β-diol-12 en-28-oic acid 3-*O*-β-d-glucosyl-(1 → 4)-β-d-glucoside	T4	B	[81, 84]
**38**	Arjunetoside	T4	R, SB	[82]
**39**	Olean 3β,6β,22α-triol-12en-28-oic acid-3-*O*-β-d-glucosyl-(1 → 4)-β-d-glucoside	T4	B	[84]
**40**	2α,19α,Dihydroxy-3-oxo-olean-12-en-28-oic acid-28-*O*-β-d-glucoside	T4	R	[85]
**41**	Ivorengenin A (2α,19α,24-trihydroxy-3-oxoolean-12-en-28-oic acid)	T28	B	[132]
**42**	Chebuloside I	T17	F	[115]
**43**	Chebuloside II	T17 T32	F B	[115] [138]
**44**	Arjunglucoside	T17 T44 T33	F R, SB SB	[115] [133] [158]
**45**	Glaucescic acid (2α,3α,6α,23-tetrahydroxyolean-2-en-28-oic acid)	T25	R	[69]
**46**	Glaucinoic acid (2α,3β,19α,24-tetrahydroxyolean-12-en-30-oic acid)	T25	SB	[130]
**47**	Termiarjunoside I (olean-1α,3β,9α,22α-tetraol-12-en-28-oic acid-3-β-d-glucoside)	T4	SB	[156]
**48**	Termiarjunoside II (olean-3α,5α,25-triol-12-en-23,28-dioic acid-3α-d-glucoside)	T4	SB	[156]
**49**	β-Amyrin	T25 T36	SB B	[129] [140]
**50**	Ivorenoside A	T28	B	[131]
**51**	Ivorenoside B	T28	B	[131]
**52**	Ivorenoside C	T28	B	[131]
**53**	Ivorengenin B (4-oxo-19α-hydroxy-3,24-dinor-2,4-secoolean-12-ene-2,28-dioic acid)	T28	B	[132]
**54**	1α,3β-Hydroxyimberbic acid 23-*O*-α-l-4-acetylrhamnoside	T47	SB	[48]
**55**	1α,3β,3,23-Trihydroxy-olean-12-en-29-oate-23-*O*-α-[4-acetoxyrhamnosyl]-29-α-rhamnoside	T47	SB	[48]
**56**	2α,3β-Dihydroxyolean-12-en-28-oic acid 28-*O*-β-d-glucoside	T48	SB	[49]
**57**	2α,3β,21β-Trihydroxyolean-12-en-28-oic acid 28-*O*-β-d-glucoside	T48	SB	[49]
**58**	2α,3β,29-Trihydroxyolean-12-en-28-oic acid 28-*O*-β-d-glucoside	T48	SB	[49]
**59**	2α,3β,23,27-Tetrahydroxyolean-12-en-28-oic acid 28-*O*-β-d-glucoside	T48	SB	[49]
**60**	Terminaliaside A ((3β,21β,22α)-3-*O*-(3′-*O*-angeloylglucosyl)-21,22-dihydroxy-28-*O*-sophorosyl-16-oxoolean-12-ene)	T50	R	[72]
**61**	2, 3, 23-Trihydroxylolean-12-ene	T7	RB	[91]
**62**	2α,3β,23-Trihydroxylolean-12-en-28-oic acid	T48	SB	[49]
**63**	23-*O*-galloylpinfaenoic acid 28-*O*-β-d-glucosyl ester	T17	F	[146]
**64**	Pinfaenoic acid 28-*O*-β-d-glucosyl ester	T4 T17	B F	[76] [146]
**65**	2α,3β-Dihydroxyurs-12,18-dien-28-oic acid 28-*O*-β-d-glucosyl ester	T4	B	[76]
**66**	Quadranoside VIII	T4	B	[76]
**67**	Kajiichigoside F1	T4	B	[76]
**68**	2α,3β,23Trihydroxyurs-12,19-dien-28-oic acid 28-*O*-β-d-glucosyl ester	T4	B	[76]
**69**	α-Amyrin	T7	RB	[91]
**70**	2α,3β,23-Trihydroxy-urs-12-en-28-oic acid	T34	L	[35]
**71**	2α-Hydroxyursolic acid	T34 T17	L F	[35] [115, 116]
**72**	Ursolic acid	T11	L	[35]
**73**	2α-Hydroxymicromeric acid	T17	F	[115, 116]
**74**	Betulinic acid	T1 T11 T12 T4, T16, T17, T20, T39 T8 T25 T28 T36	B L B B, L, S, R, F B, L, S, R SB B B	[55] [35] [99] [23] [23] [129] [132] [140]
**75**	Terminic acid	T4	R, H	[57, 62]
**76**	Lupeol	T4 T25 T44	SB SB SB, R	[80] [129] [43]
**77**	Monogynol A	T12	B	[99]
**78**	Triterpenes	T25 T44	SB R, SB	[129] [133]
**79**	Friedelin	T4 T7 T25 T34	F RB SB SB	[83] [93] [129, 130] [35]
**80**	Maslinic lactone	T1	H	[56]
**81**	Terminalin A	T25	SB	[129]
**82**	Arjunaside A	T4	B	[68]
**83**	Arjunaside B	T4	B	[68]
**84**	Arjunaside C	T4	B	[68]
**85**	Arjunaside D	T4	B	[68]
**86**	Arjunaside E	T4	B	[68]
Mono- (14) and sesqui- (4) terpendoids				
**87**	α-Pinene	T9	L	[13]
**88**	Sabinene	T9	L	[13]
**89**	Myrcene	T9	L	[13]
**90**	β-Pinene	T9	L	[13]
**91**	1,8-Cineole	T9	L	[13]
**92**	Linalool	T9	L	[13]
**93**	Menthone	T9	L	[13]
**94**	γ-Terpineol	T9	L	[13]
**95**	α-Terpineol	T9	L	[13]
**96**	Limonene	T9	L	[13]
**97**	Neral	T9	L	[13]
**98**	Geraniol	T9	L	[13]
**99**	Thymol	T9	L	[13]
**100**	Isomenthone	T9	L	[13]
**101**	β-Copaene	T9	L	[13]
**102**	β-Caryophyllene	T9	L	[13]
**103**	Caryophyllene	T9	L	[13]
**104**	α-Humulene	T9	L	[13]
Hydrolysable (89) and condensed tannins (2)				
**105**	1,2,3,6-Tetra-*O*-galloyl-β-d-glucose	T17	F	[159]
**106**	Gallotannin (1,2,3,4,6 penta galloyl glucose)	T4 T17 T19 T30 T45, T46	SB, L F F R L	[86] [21, 118, 119] [120] [133] [133]
**107**	1,3,4,6-Tetra-*O*-galloyl-β-d-glucose	T17	F	[159]
**108**	2,3,4,6-Tetra-*O*-galloyl-d-glucose	T3 T4	F SB, L	[154] [86]
**109**	1,2,6-Tri-*O*-galloyl-β-d-glucose	T31	R	[101]
**110**	Sanguiin H-1	T14	L	[102]
**111**	1,6-Di-*O*-galloyl-β-d-glucose	T3 T17 T40	F F B	[154] [21, 119] [41]
**112**	1,3,6-Tri-*O*-galloyl-β-d-glucose	T3 T40 T19 T17	F B F F	[154] [41] [120] [159]
**113**	Methyl 3,6-di-*O*-galloyl-β-d-glucoside	T40	B	[41]
**114**	4,6 Bis hexahydroxydiphenyl-1-galloyl-glucose	T4	SB, L	[86]
**115**	Sanguiin H-4	T14	L	[18, 102]
**116**	Corilagin	T3 T31 T16 T17 T19 T24 T32	F R L, B F F F L	[154] [101] [41, 106, 107] [21, 118, 119, 159] [120] [126] [135, 136]
**117**	Tercatain	T16 T17	B, L F	[41, 106, 107] [159]
**118**	1,3-Di-*O*-galloyl-β-d-glucose	T17	F	[159]
**119**	2,3-*O*-(*S*)-HHDP-d-glucose	T3 T14 T4 T16 T40 T36	F L B B, L B L	[154] [102] [104] [41, 107] [41] [38]
**120**	2,3-(*S*)-HHDP-6-*O*-galloyl-d-glucose	T3 T4 T40 T32	F B B B	[154] [104] [41] [137]
**121**	3,6-Di-*O*-galloyl-d-glucose	T3 T40 T17	F B F	[154] [41] [159]
**122**	3,4-Di-*O*-galloyl-d-glucose	T3	F	[154]
**123**	6-*O*-galloyl-d-glucose	T17	F	[159]
**124**	3,4,6-Tri-*O*-galloyl-d-glucose	T17	F	[159]
**125**	Tellimagrandin I	T35 T17	L F	[139] [159]
**126**	Gemin D	T17	F	[159]
**127**	Arjunin	T4 T17	L F	[65, 86] [115]
**128**	Punicalin	T3 T4 T14 T40 T16 T17 T28 T49	F L, B L B L L, F SB L	[154] [65, 86, 104] [102] [41] [106, 107] [21, 155] [29] [149]
**129**	Casuarinin	T4 T16 T17	L, B B F	[88, 104] [41] [21, 118, 119]
**130**	Casuariin	T4	B	[90, 104]
**131**	Terchebulin	T3 T4 T7 T12 T17 T31	F B SB B F W	[154] [90, 104] [92] [100] [21] [134]
**132**	Castalagin	T4 T16, T40	B B	[90, 104] [41]
**133**	Grandinin	T16, T40	B	[41]
**134**	Castalin	T16, T40	B	[41]
**135**	α/β-Punicalagin	T3 T7 T4 T11 T12 T31 T14 T16 T17 T40 T19 T28 T32 T35 T36 T38	F SB B L B R L B L, F B F SB B L L L	[154] [92] [104] [35] [100] [101] [18, 103] [41] [21, 106, 119, 155] [41] [120] [29] [137] [139] [38] [39]
**136**	1-α-*O*-galloylpunicalagin	T14	L	[18, 102, 103]
**137**	6′-*O*-methyl neochebulagate	T17	F	[159]
**138**	Dimethyl neochebulagate	T17	F	[159]
**139**	Neochebulagic acid	T17	F	[159]
**140**	Dimethyl 4′-epi-neochebulagate	T17	F	[159]
**141**	Methyl chebulagate	T17	F	[159]
**142**	Chebulagic acid	T3 T4 T8 T17 T16 T39 T20 T19 T32 T35	F B, L, S F, B, L, S F, B, L, S, R F, B, L, S, R F, B, L, S, R F, B, L, R F L L	[154] [23] [23] [23, 96] [3, 4, 9, 21, 110] [23] [23] [120] [135, 136] [139]
**143**	Chebulinic acid	T3 T4, T8, T16, T20, T39 T17 T32 T35	F F, B, L, S, R F, B, L, S, R L L	[154] [23] [3, 4, 21, 110, 119, 155] [23] [110, 135, 139]
**144**	Chebulanin	T34, T11 T17	L F	[35] [21, 119, 155, 159]
**145**	1,3-Di-*O*-galloyl-2,4-chebuloyl-β-d-glucose	T3	F	[154]
**146**	1,6-Di-*O*-galloyl-2,4-chebuloyl-β-d-glucose	T17	F	[155, 159]
**147**	2-*O*-galloylpunicalin	T14 T40 T32 T49	L B B L	[18] [41] [137] [149]
**148**	1-Desgalloyleugeniin	T14 T16	L L	[102] [107]
**149**	Eugeniin	T14	L	[102]
**150**	Rugosin A	T14	L	[102]
**151**	1(α)-*O*-galloylpedunculagin	T14	L	[102]
**152**	Praecoxin A	T14	L	[102]
**153**	Calamansanin	T14	L	[102]
**154**	Calamanin A	T14	L	[102]
**155**	Calamanin B	T14	L	[102]
**156**	Calamanin C	T14	L	[102]
**157**	Terflavin C	T4 T14 T17	B L L	[104] [103] [21]
**158**	Terflavin A	T16 T17 T32	L F B	[106, 107] [21] [137]
**159**	Terflavin B	T16 T17 T32	L L, F B	[106, 107] [21, 155] [137]
**160**	3-Methoxy-4-hydroxyphenol-1-*O*-β-d-(6′-*O*-galloyl)-glucoside	T16	B	[41]
**161**	3,5-Di-methoxy-4-hydroxyphenol-1-*O*-β-d-(6′-*O*-galloyl)-glucoside	T16	B	[41]
**162**	Acutissimin A	T16	B	[41]
**163**	Eugenigrandin A	T16	B	[41]
**164**	Catappanin A	T16	B	[41]
**165**	Castamollinin	T40	B	[41]
**166**	Tergallagin	T16	L	[106, 107]
**167**	Geraniin	T16	L	[107]
**168**	Granatin B	T16	L	[107]
**169**	Gallotannic (tannic acid)	T17,T8 T38	F L	[113] [141]
**170**	Chebulin	T17	F	[113, 114]
**171**	Terchebin	T17	F	[113, 119]
**172**	Neochebulinic acid	T3 T17	F F	[154] [21, 119, 155]
**173**	Chebumeinin A	T17	F	[118]
**174**	Chebumeinin B	T17	F	[118]
**175**	Isoterchebulin	T32	B	[137]
**176**	Punicacortein C	T3 T32 T17	F B F	[154] [137] [159]
**177**	Punicacortein D	T17	F	[159]
**178**	4,6-*O*-Isoterchebuloyl-d-glucose	T32	B	[137]
**179**	Trigalloyl-β-d-glucose	T35	L	[139]
**180**	Tetragalloyl-β-d-glucose	T35	L	[139]
**181**	Pentagalloyl-β-d-glucose	T35	L	[139]
**182**	1,2,3-Tri-*O*-galloyl-6-*O*-cinnamoyl-β-d-glucose	T17	F	[159]
**183**	1,2,3,6-Tetra-*O*-galloyl-4-*O*-cinnamoyl-β-d-glucose	T17	F	[159]
**184**	1,6-Di-*O*-galloyl-2-*O*-cinnamoyl-β-d-glucose	T17	F	[159]
**185**	1,2-Di-*O*-galloyl-6-*O*-cinnamoyl-β-d-glucose	T17	F	[159]
**186**	4-*O*-(2′′, 4′′-di-*O*-galloyl-α-l-rhamnosyl) ellagic acid	T17	F	[159]
**187**	4-*O*-(4′′-*O*-galloyl-α-l-rhamnosyl) ellagic acid	T17	F	[159]
**188**	4-*O*-(3′′, 4′′-di-*O*-galloyl-α-l-rhamnosyl) ellagic acid	T17	F	[159]
**189**	1′-*O*-methyl neochebulanin	T17	F	[159]
**190**	Dimethyl neochebulinate	T17	F	[159]
**191**	Phyllanemblinin E	T17	F	[159]
**192**	1′-*O*-methyl neochebulinate	T17	F	[159]
**193**	Phyllanemblinin F	T17	F	[159]
**194**	Procyanidin B-1	T16	B	[41]
**195**	3′-*O*-galloyl procyanidin B-2	T16	B	[41]
Flavonoids (45)				
**196**	5,7,2′-Tri-*O*-methylflavanone4′-*O*-α-l-rhamnosyl-(1 → 4)-β-d-glucoside	T1	R	[52]
**197**	Arjunone	T4	B, F	[83, 89]
**198**	8-Methyl-5,7,2′,4′-tetramethoxy-flavanone	T1 T39	R B	[53] [144]
**199**	Naringin	T4 T8 T17 T39 T20	L, S, F B, F L, R, F R, F B, L, S, R	[23] [23] [23] [23] [23]
**200**	Eriodictyol	T4, T8, T17, T20, T39 T16	B, L, S, R, F L, S, R, F	[23] [23]
**201**	Hesperitin	T24	F	[122]
**202**	Flavanone	T24	F	[122]
**203**	Arjunolone (6,4-dihydroxy-7-methoxy flavone)	T4	SB	[64]
**204**	Bicalein (5,6,7-trihydroxy flavone)	T4	SB	[64]
**205**	Scutellarein	T4 T8, T17, T20 T16 T39	B, R B, L, S, R, F L, F B, L, R, F	[23] [23] [23] [23]
**206**	Luteolin	T4 T8, T20 T17 T16 T39 T24	B, L L, S R, L L L, S, F F	[23, 65] [23] [23] [23] [23] [122]
**207**	Apigenin	T4 T8, T16, T17, T20, T39	B, L, S, R, F B, L, S, R, F	[23, 66] [23]
**208**	Isoorientin	T11 T4, T8, T17, T16, T20, T39 T35 T36	L B, L, S, R, F L L	[35] [23] [139] [38]
**209**	Orientin	T11 T4 T8 T17 T16 T39 T20 T35 T36	L L, F B, S B, L, S, R, F L, R, F B, S, F L, S, F, R L L	[35] [23] [23] [23] [23] [23] [23] [139] [38]
**210**	Isovitexin	T11 T4 T17 T16 T39 T20 T35 T36	L L, F L, R, F L S, F L, S, F L L	[35] [23] [23] [23, 105] [23] [23] [139] [38]
**211**	Apigenin-6-C-(2″-*O*-galloyl)-β-d-glucoside	T16	L	[105]
**212**	Apigenin-8-C-(2″-*O*-galloyl)-β-d-glucoside	T16 T34	L L	[105] [35]
**213**	Vitexin	T4, T17, T20 T8 T16 T39 T35 T36	B, L, S, R, F B, L, S, R L, S, R, F B, L, S, F L L	[23] [23] [23] [23] [139] [38]
**214**	Amentoflavone	T8 T17 T20	L, S L, R, F L	[23] [23] [23]
**215**	Neosaponarin	T36	L	[38]
**216**	(−)-Epicatechin	T4	B	[76]
**217**	Epicatechin	T4, T8, T17, T20, T39 T16 T34	B, L, S, R, F L, S, R, F SB	[23] [23] [35]
**218**	Catechin	T34 T11 T4, T8, T16, T17, T20, T39 T44	SB L B, L, S, R, F R	[35] [35] [23] [133]
**219**	Catechin–epicatechin	T44	R	[43]
**220**	Catechin–epigallocatechin	T44	R	[43]
**221**	Epigallocatechin	T34	SB	[35]
**222**	(−)-Epicatechin-3-*O*-gallate	T16	B	[41]
**223**	(−)-Epigallocatechin-3-*O*-gallate	T16	B	[41]
**224**	Flavanol	T24	F	[122]
**225**	Gallocatechin	T34 T24	SB F	[35] [126]
**226**	Quercetin	T4 T8 T17 T16 T39 T20 T24 T49	B, L, R R S, R, F L, S, F L, B F F L	[23] [23] [23] [23] [23, 142] [23] [124] [124]
**227**	Kaempferol	T4 T8 T16, T17 T20, T39 T24	B, L, S, R, F B, L, S, F B, L, S, R, F L, S, R, F F	[23, 66] [23] [23] [23] [122]
**228**	Kaempferol-3-*O*-β-d-rutinoside	T4, T8, T17 T16 T39 T20 T36	B, L, S, R, F L, S, F L, R, F L, S, R L	[23] [23] [23] [23] [38]
**229**	Afzelin (kaempferol 3-*O*-rhamnoside)	T49	L	[124]
**230**	Rutin	T4, T16 T8 T17, T39 T20 T32 T36	B, L, S, F L, S B, L, S, R, F L, S, F L L	[23] [23] [23] [23] [135, 136] [38]
**231**	Narcissin	T32	L	[135, 136]
**232**	Quercetin-3,4′-di-*O*-glucoside	T4 T8 T16, T17, T20, T39	B, L, S, F B, S, F B, L, S, R, F	[23] [23] [23]
**233**	Quercetin-7-*O*-rhamnoside	T4	F	[80]
**234**	2-*O*-β-glucosyloxy-4,6,2′,4′-tetramethoxychalcone	T1	R	[53]
**235**	Cerasidin	T4	F	[80]
**236**	Genistein	T4 T8, T16, T17, T20, T39	B, L, S, R, F B, L, S, R, F	[23, 80] [23]
**237**	Cyaniding	T4	B	[66]
**238**	Pelargonidin	T4	B	[66]
**239**	Leucocyanidin	T4	B	[80]
**240**	7-Hydroxy-3′,4-(methylenedioxy)flavan	T8	FR	[12]
Lignan (27)				
**241**	Termilignan	T8 T39	FR B	[12] [144]
**242**	Anolignan B	T8 T44	FR R	[12] [43, 151]
**243**	Thannilignan	T8	FR	[12]
**244**	Termilignan B	T44	R	[133]
**245**	Ferulic acid dehydrodimer	T24	F	[125]
**246**	(7*S*,8*R*,7′*R*,8′*S*)-4′-hydroxy-4-methoxy-7,7′-epoxylignan	T48	SB	[50]
**247**	Meso-(rel7*S*,8*R*,7′*R*,8′*S*)-4,4′-dimethoxy-7,7′-epoxylignan	T48	SB	[50]
**248**	4′-*O*-cinnamoyl cleomiscosin A	T50	R	[72]
**249**	Diethylstilbestrol monosulphate	T24	F	[126]
**250**	Terminaloside A	T19	L	[22]
**251**	Terminaloside B	T19	L	[22]
**252**	Terminaloside C	T19	L	[22]
**253**	Terminaloside D	T19	L	[22]
**254**	Terminaloside E	T19	L	[22]
**255**	Terminaloside F	T19	L	[22]
**256**	Terminaloside G	T19	L	[22]
**257**	Terminaloside H	T19	L	[22]
**258**	Terminaloside I	T19	L	[22]
**259**	Terminaloside J	T19	L	[22]
**260**	Terminaloside K	T19	L	[22]
**261**	2-Epiterminaloside D	T19	L	[22]
**262**	6-Epiterminaloside K	T19	L	[22]
**263**	Terminaloside L	T19	L	[121]
**264**	Terminaloside M	T19	L	[121]
**265**	Terminaloside N	T19	L	[121]
**266**	Terminaloside O	T19	L	[121]
**267**	Terminaloside P	T19	L	[121]
Phenols and glycosides (52)				
**268**	Ellagic acid	T1 T7 T10, TM, TT T12 T40 T4, T8, T20 T17 T16 T39 T24 T25 T31 T28, T32 T35 T42 T30, T44 T36, T45, T46 T48 T49	B SB SB B B B, L, S, R, F L, SB, R F SB, L, R, F B, L, S, R, F, H F B, R, Rl B H L, F R, SB R L SB L	[55] [92, 127] [14] [100] [41] [23, 80, 83, 86] [3, 9, 21, 23, 111, 119] [14, 23, 41, 108, 144] [23, 142] [123] [70, 127, 128] [127, 134] [128] [37, 38] [133] [133] [133] [50] [124]
**269**	Methyl ellagic acid	T4	B	[90]
**270**	3-*O*-methylellagic acid	T33	SB	[158]
**271**	3,3′-Di-*O*-methylellagic acid	T28 T39 T48	SB H,B SB	[29] [8, 9, 143, 144] [50]
**272**	3,3′-Di-*O*-methylellagic acid 4-mono glucoside	T39	H	[147, 148]
**273**	Tetra-*O*-methyl ellagic acid	T39	H	[148]
**274**	3,3′-Di-*O*-methylellagic acid 4-*O*-β-d-glucosyl-(1 → 4)-β-d-glucosyl-(1 → 2)-α-l-arabinoside	T1	R	[52]
**275**	3,4,3′-Tri-*O*-methylflavellagic acid	T7 T12 T24 T25 T31 T28 T32 T39	B B F L, B, R, Rl B SB, H H, B H	[126] [100] [126] [26, 70, 127, 128] [127] [29, 128] [128, 138] [143, 148]
**276**	3,3′,4-*O*-trimethyl-4′-*O*-β-d-glucosylellagic acid	T28	SB	[29]
**277**	3,3′-Di-*O*-methyl ellagic acid 4′-*O*-β-d-xyloside	T48	SB	[50]
**278**	3,4′-Di-*O*-methylellagic acid 3′-*O*-β-d-xyloside	T48	SB	[153]
**279**	4′-*O*-galloy-3,3′-di-*O*-methylellagic acid 4-*O*-β-d-xyloside	T48	SB	[153]
**280**	Flavogallonic acid	T7 T40 T31 T12 T36	SB B W R L	[92] [41] [134] [101] [38]
**281**	Methyl (*S*)-flavogallonate	T36	L	[38]
**282**	Vanillic acid 4-*O*-β-d-(6′-*O*-galloyl) glucoside	T32	B	[138]
**283**	3-*O*-methylellagic acid 4′-*O*-α-l-rhamnoside	T4 T34 T33	B SB SB	[76] [35] [158]
**284**	Eschweilenol C (ellagic acid 4-*O*-α-l-rhamnoside)	T12 T17	B F	[100] [164]
**285**	3-*O*-methylellagic acid 4′-*O*-xyloside	T31	R	[101]
**286**	Brevifolincarboxylic acid	T35	L	[139]
		T17	F	[159]
**287**	Terflavin D	T17	L	[21]
**288**	Gallic acid	T3 T4, T8, T20, T39 T10, TM, TT T17 T16 T34 T12 T31 T40 T24 T30 T35 T36 T38 T42 T44 T45, T46 T48 T49	F B, L, S, R, F SB SB, F, R, L SB, F, R, L L B R, W B F R L L L R, SB R L SB L	[154] [23, 80, 83, 86] [14] [14, 21, 23, 118, 119] [14, 23, 41, 108] [35] [100] [101, 134] [41] [123, 125] [133] [139] [38] [141] [133] [133] [133] [50] [124]
**289**	Phyllemblin (ethyl gallate isomers1 progallin A)	T4 T8 T24 T28 T36	B F F SB L	[86] [96, 113] [126] [29] [38]
**290**	Monogalloyl glucose	T3 T8 T17 T31	F F F R	[154] [113] [21] [101]
**291**	Methyl gallate	T14 T8 T32 T36 T48 T49	L F L L SB L	[18] [113] [135, 136] [38] [50] [124]
**292**	Shikimic acid	T32	L	[135, 136]
**293**	5-*O*-galloyl-(−)-shikimic acid	T3 T17	F F	[118] [154, 159]
**294**	4-*O*-galloyl-(−)-shikimic acid	T17	F	[159]
**295**	3,5-Di-*O*-galloyl-(−)-shikimic acid	T3	F	[154]
**296**	Digallic acid	T17	F	[159]
**297**	Ethyl gallate isomers2	T24	F	[126]
**298**	Ethyl gallate isomers3	T24	F	[126]
**299**	Dimethyl gallic acid	T35	L	[139]
**300**	Chebulic acid	T3 T17 T24 T35	F F F L	[154] [4, 9, 112, 119, 159] [125, 126] [139]
**301**	6′-*O*-methyl chebulate	T17	F	[159]
**302**	7′-*O*-methyl chebulate	T17	F	[159]
**303**	Chebulic acid trimethyl ester	T32	L	[135, 136]
**304**	Terminalin	*T38*	L	[39]
**305**	Decarboxyellagic acid	T3	F	[154]
**306**	3-*O*-galloyl-d-glucose	T3	F	[154]
**307**	6-*O*-galloyl-d-glucose	T3 T17	F F	[154] [159]
**308**	Vanillic acid	T4, T8, T20, T39 T17 T16 T44	B, L, S, R, F B S, R, B, F R	[23] [23, 117] [23] [43]
**309**	Benzoic acid	T44 T24	R F	[43] [122]
**310**	Hydrocinnamic acid	T44	R	[43]
**311**	Gentisic acid	T16	L	[108]
**312**	Protocatechuic acid	T4, T8, T16, T17, T20, T39	B, L, S, R, F	[23]
**313**	2,3-Di-hydroxyphenyl β-d-glucosiduronic acid	T24	F	[125]
**314**	Quinic acid	T4, T8, T16, T17, T20, T39 T24	B, L, S, R, F	[23] [125]
**315**	*p*-Coumaric acid	T17 T44	WP R	[117] [43]
**316**	Caffeic acid	T4, T8 T17 T16 T39 T20 T44	L, S L, S, R L B, L, S, R, F B R	[23] [23] [23] [23] [23] [43]
**317**	Chlorogenic acid	T4 T17 T16, T39 T20	L, S S, R, F, L L B	[23] [23] [23] [23]
**318**	Ferulic acid	T4 T8, T17, T20, T39 T16	B, L, S, F B, L, S, R, F L, S, R	[23] [23] [23]
**319**	Sinapic acid	T4, T16, T20, T39 T8 T17	B, L, S, R, F S, R, F B, S, R, F	[23] [23] [23]
Steroids (8), polyols (9) and esters (6)				
**320**	β-Sitosterol	T1 T4 T8 T12 T16 T48 T25 T36 T39 T44	B, H S, F F F B, SB H H SB B H, SB, R	[55, 56] [57, 83] [96, 113] [99] [128] [128] [129] [140] [147, 148] [43, 133, 152]
**321**	β-Sitosterol-3-acetate	T44	SB, R	[43]
**322**	β-Sitosteryl palmitate	T16 T25, T31	SB, H L,F	[128] [128]
**323**	Stigmasterol 3-*O*-β-d-glucoside	T4 T33	F SB	[80] [158]
**324**	Stigmasterol	T12 T25 T33 T44	B SB SB RB	[99] [129] [158] [133, 152]
**325**	Stigma-4-ene-3-one	T44	RB	[43]
**326**	16,17-Dihydroneridienone 3*O*-β-d-glucosyl-(1 → 6)-*O*-β-d-galactoside	T4	R	[59]
**327**	Cannogenol 3-*O*-β-d-galactosyl-(1 → 4)-*O*-α-l-rhamno-side	T8	Se	[94]
**328**	2-Hexanol	T9	L	[13]
**329**	Octanol	T9	L	[13]
**330**	Methoxycarbonyloxymethyl methylcarbonate	T24	F	[125]
**331**	Ribonolactone	T24	F	[125]
**332**	Apionic acid	T24	F	[125]
**333**	Ascorbic acid	T24	F	[125]
**334**	Gluconolactone	T24	F	[125]
**335**	Glucohepatonic acid-1,4-lactone	T24	F	[125]
**336**	Galacturonic acid	T44	R	[43]
**337**	Geranyl formate	T9	L	[13]
**338**	Citronellyl acetate	T9	L	[13]
**339**	Geranyl acetate	T9	L	[13]
**340**	Geranyl tiglate	T9	L	[13]
**341**	Laxiflorin	T31	RB	[127]
**342**	(1*S*,5*R*)-4-oxo-6,8-dioxabicyclo[3.2.1]oct-2-ene-2-carboxylic acid	T24	F	[125]
Others (26)				
**343**	Glucuronic acid	T24	F	[125]
**344**	Coumarin	T45	L	[133]
**345**	Eujavonic acid	T24	F	[125]
**346**	Purine	T24	F	[125]
**347**	5-(4-Hydroxy-2,5-dimethylphenoxy)-2,2-dimethylpentanoic acid (gemfibrozil M1)	T24	F	[125]
**348**	*p*-Hydroxytiaprofenic acid	T24	F	[125]
**349**	*Cis*-polyisoprene	T32	L	[135]
**350**	Arachidic acid	T17	F	[113]
**351**	Behenic acid	T8, T17	F	[113]
**352**	Arjunaphthanoloside	T4	SB	[87]
**353**	Resveratrol (3′,4,5′-trihydroxystilbene)	T24 T44	F R	[126] [43]
**354**	Resveratrol glucoside (piceid)	T24 T44	F RB	[126] [152]
**355**	Resveratrol-β-d-glucoside	T44	RB	[152]
**356**	Combretastatin	T24	F	[126]
**357**	Combretastatin A1	T24	F	[126]
**358**	(*Z*)-Stilbene	T44	R	[133]
**359**	(*E*)-Stilbene	T44	R	[133]
**360**	3′5′-Dihydroxy-4-(2-hydroxyethoxy) resveratrol-3-*O*-β-rutinoside	T44	R, RB	[43, 152]
**361**	Resveratrol-3-β-rutinoside glycoside	T44	R, RB	[43, 152]
**362**	1,4-Cineole	T9	L	[13]
**363**	Terpinen-4-ol	T9	L	[13]
**364**	Terminalianone	T12	B	[98]
**365**	Termicalcicolanone A	T15	WP	[19]
**366**	Termicalcicolanone B	T15	WP	[19]
**367**	Mangiferin	T4 T8 T17 T16 T39 T20	B, S, F B, R, F B, L, S, R, F L, R, F B, L, S, F L, S, R	[23] [23] [23] [23] [23] [23]
**368**	Benzoyl-β-d-(4′ → 10″geranilanoxy)-pyranoside	T8	F	[160]

*R* root, *SB* stem bark, *B* bark, *F* fruit, *S* stem, *H* heartwood, *RB* root bark, *Rl* rootlet, *Se* seed, *FR* fruit rind, *WP* whole plant, *T1*–*T50* plants from Table 1, *TM T. manii*, *TT T. tomentosa*

**Table 3:** The numbers and main types of compounds reported from different *Terminalia* species

No.	Plant	Plant organs	Numbers	Main types
T1	*T. alata*	Roots, barks	18	Triterpenes
T3	*T. arborea*	Fruits	24	Hydrolysable tannin
T4	*T. arjuna*	Whole plants	93	Triterpenes, tannins, flavonoids
T7	*T. avicennioides*	Barks	10	Triterpenes, tannins
T8	*T. bellirica*	Fruits, barks	45	Triterpenes, flavonoids, lignin, simple phenols
T9	*T. bentzoe*	Leaves	29	Monoterpenoids, sesquiterpenoid
T11	*T. brachystemma*	Leaves	8	Flavonoids
T12	*T. brownii*	Leaves	13	Triterpenes
T14	*T. calamansanai*	Leaves	18	Hydrolysable tannin
T16	*T. catappa*	Whole plants	64	Triterpenes, tannins, flavonoids, simple phenols
T17	*T. chebula*	Whole plants	120	Triterpenes, tannins, flavonoids, simple phenols
T19	*T. citrina*	Fruits, leaves	23	Lignan
T20	*T. elliptica*	Whole plants	36	Flavonoids
T24	*T. ferdinandiana*	Fruits	35	Flavonoids, simple phenols, polyols
T25	*T. glaucescens*	Barks	19	Triterpenes
T28	*T. ivorensis*	Barks	18	Triterpenes
T31	*T. laxiflora*	Roots	13	Tannins
T32	*T. macroptera*	Whole plants	28	Triterpenes, tannins, simple phenols
T33	*T. mantaly*	Stem barks	7	Triterpenes, simple phenols
T34	*T. mollis*	Barks	12	Triterpenes, flavonoids
T35	*T. muelleri*	Leaves	16	Hydrolysable tannin, flavonoids, simple phenols
T36	*T. myriocarpa*	Leaves, barks	21	Triterpenes, flavonoids, simple phenols
T39	*T. paniculata*	Barks	43	Triterpenes, flavonoids, simple phenols
T40	*T. parviflora*	Barks	16	Tannins
T44	*T. sericea*	Roots	32	Triterpenes, simple phenols, other compounds
T48	*T. superba*	Barks	15	Triterpenes, simple phenols

Chemical components identified from the other 12 species, including *T. bialata* (T10), *T. calcicola* (T15), *T. kaiserana* (T30), *T. manii* (TM), *T. macroptera* (T32), *T. oblongata* (T38), *T. sambesiaca* (T42), *T. spinosa* (T45), *T. stenostachya* (T46), *T. stuhlmannii* (T47), *T. triflora* (T49), *T. tropophylla* (T50) were less than 6 compounds

### Terpenoids

**Fig. 1:**
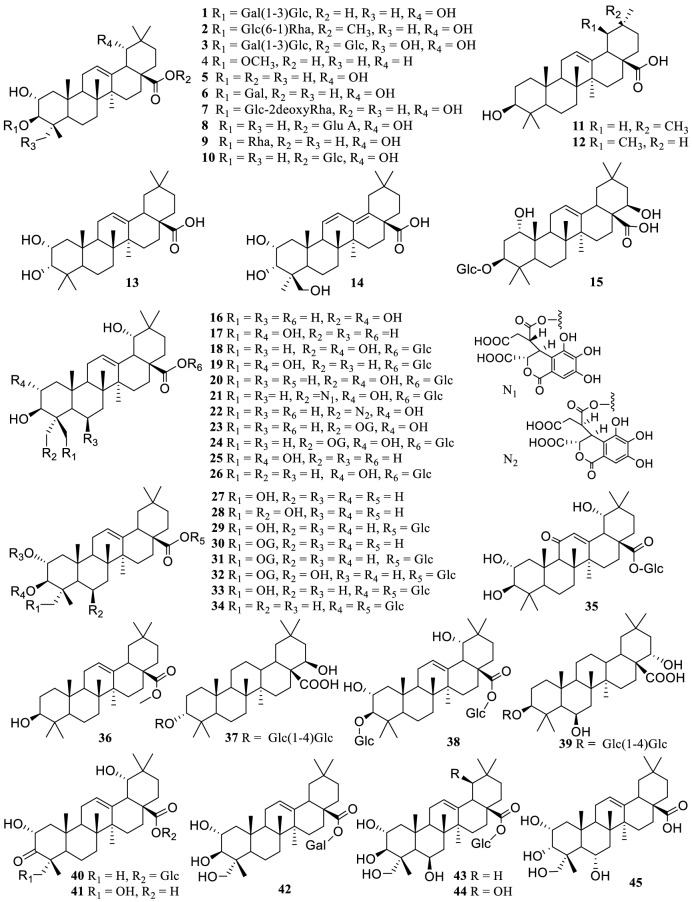

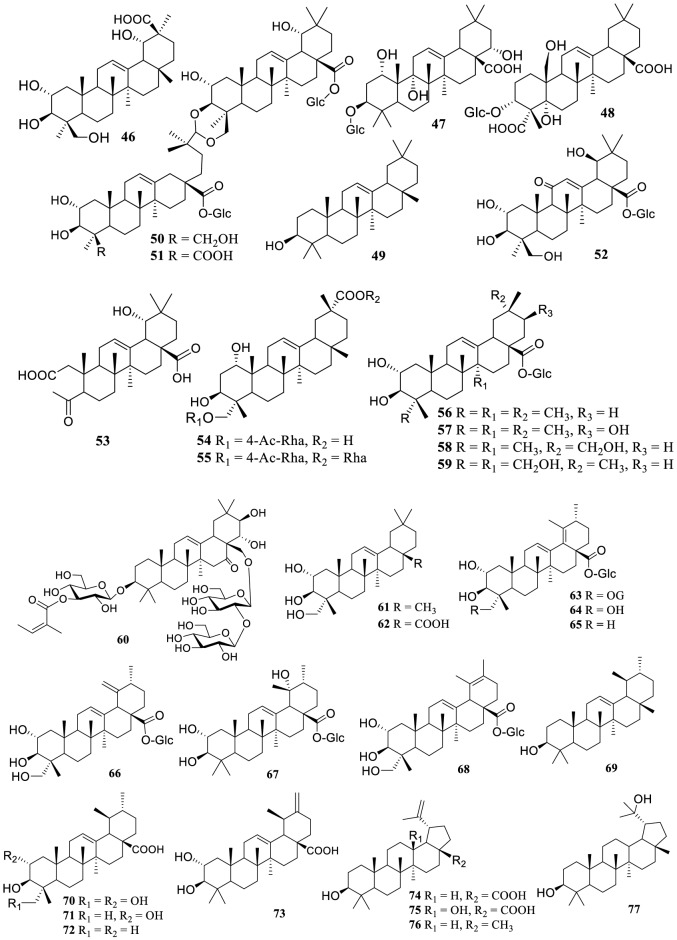

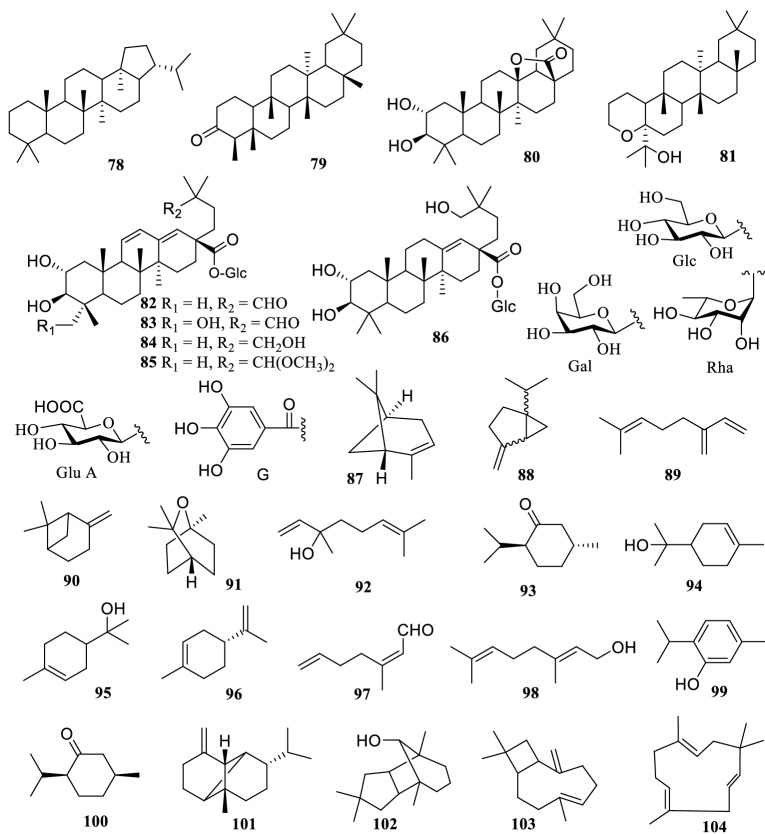
The structures of terpenoids **1–104**

So far, 104 terpenoids (Fig. 1) including 86 triterpenes (**1–86**), 14 monoterpenes (**87–100**), 4 sesquiterpenes (**101–104**) have been reported from the genus *Terminalia*. The triterpenoids are mainly oleanane, ursane and lupine types, and their glycosides. Particularly, Atta-ur-Rahman et al. isolated a new *seco*-triterpene terminalin A (**81**) possessing a novel rearranged *seco*-glutinane structure with a pyran ring-A and an isopropanol moiety from the stem barks of *T. glaucescens* [129]. Ponou et al. found two dimeric triterpenoid glucosides, ivorenosides A and B (**49–50**) possessing an unusual skeleton [131], and two new oleanane type triterpenes, 3-oxo-type ivorengenin A (**41**) and 3,24-dinor-2,4-secooleanane-type ivorengenin B (**53**) from the barks of *T. ivorensis* [132]. Compounds **41**, **49** and **53** showed significant anticancer activities. Wang et al. isolated five new 18,19-secooleanane type triterpene glycosyl esters, namely arjunasides A–E (**82–86**) from the MeOH extract of *T. arjuna*’*s* barks, TaBs [68]. Moreover, five ursane type triterpene glucosyl esters (**64–68**) were also obtained for the first time [76]. From the fruits of *T. chebula*, 23-*O*-neochebuloylarjungenin 28-*O*-β-d-glycosyl ester (**21**) and 23-*O*-4′-*epi*-neochebuloylarjungenin (**22**) with novel substituents at C-23 were reported, in addition to compounds **23–24**, **30–32** and **63**, whose C-23 substituents were gallate. Compounds **30** and **31** had strong hypoglycemic effect [146]. Furthermore, compound **40** was obtained from the barks of *T. arjuna* [85], while friedelin (**79**) with 3-oxo moiety was reported from the fruits of *T. arjuna* [83], the root barks of *T. avicennioides* [93], and the stem barks of *T. glaucescens* [130] and *T. mollis* [35].

### Tannins

**Fig. 2:**
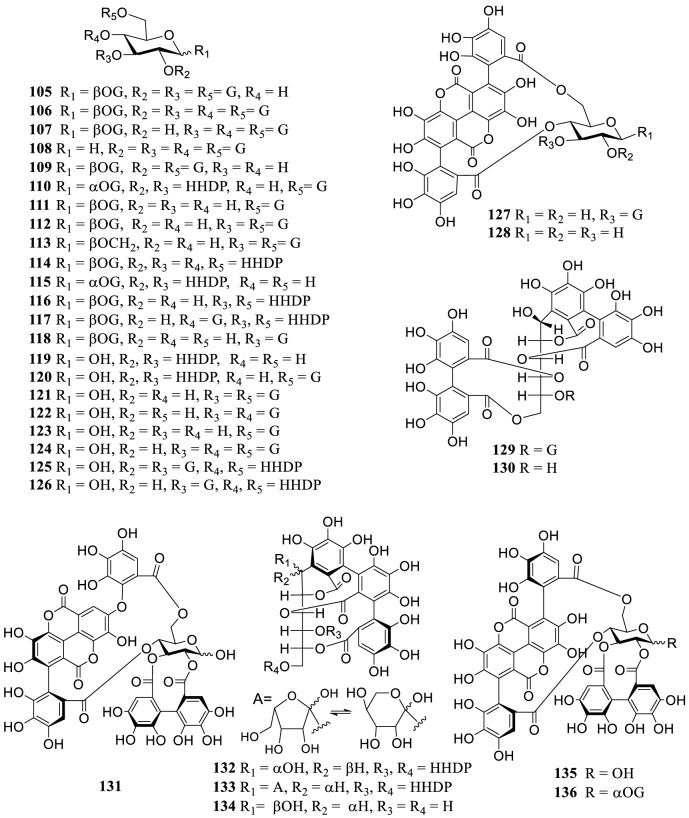

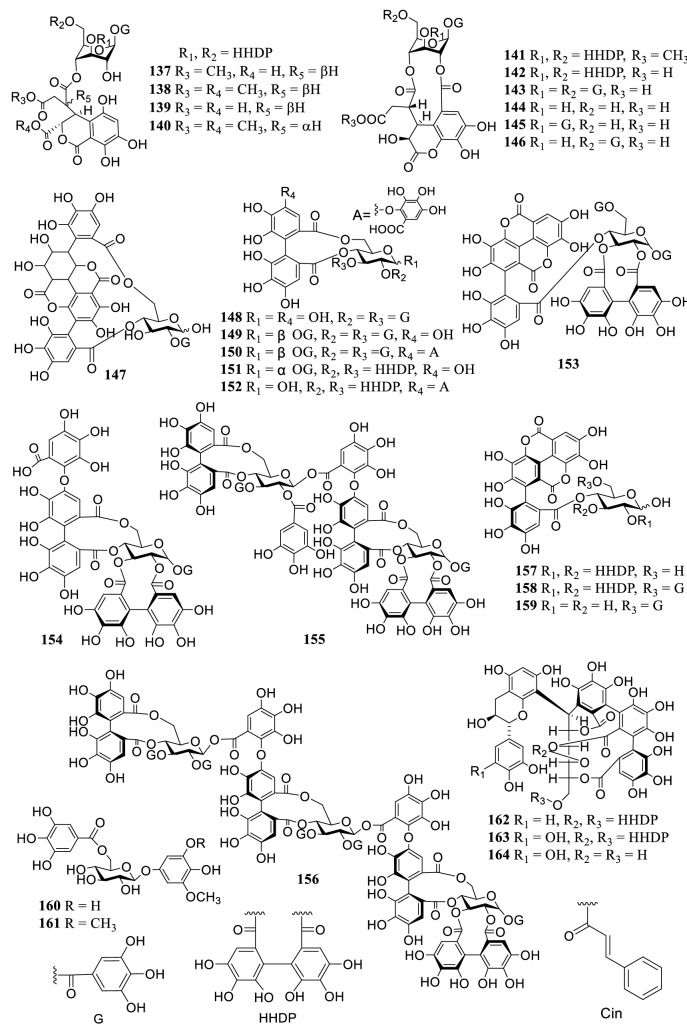

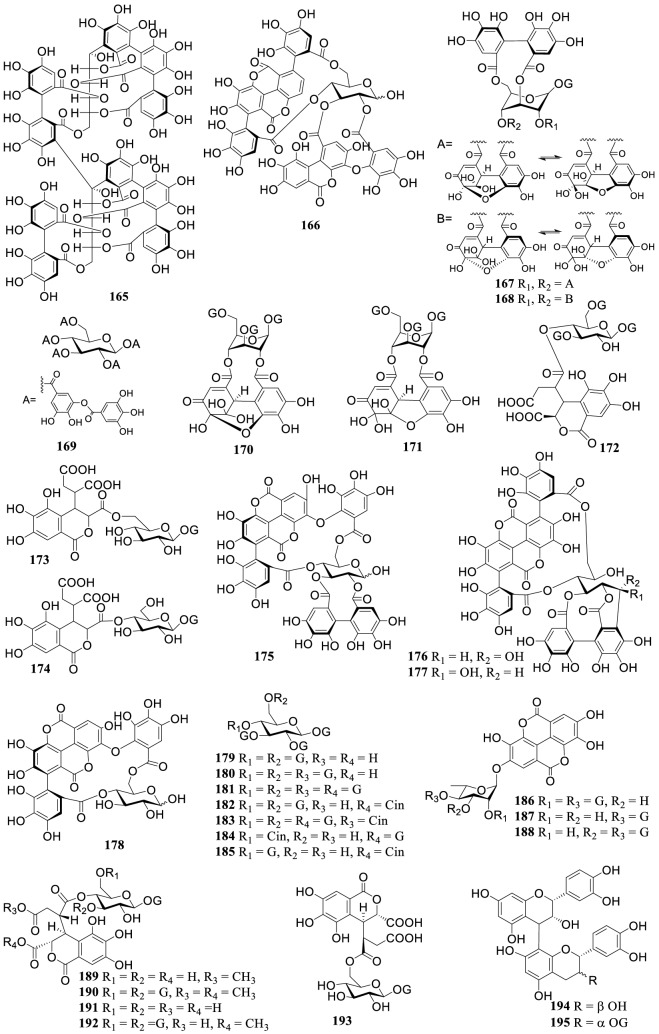
The structures of tannins **105–195**

As the main secondary metabolites, 91 tannins (**105–195**) were reported from the genus *Terminalia* (Fig. 2), including ellagitannins, gallotannins, dimeric, and trimeric tannins. Four cinnamoyl-containing gallotannins (**182–185**) were discovered firstly from the fruits of *T. chebula*, and 1,2,3,6-tetra-*O*-galloyl-4-*O*-cinnamoyl-β-d-glucose (**183**) and 4-*O*-(2″,4″-di-*O*-galloyl-α-l-rhamnosyl) ellagic acid (**186**) showed significant inhibitory activity on α-glucosidase with IC_50_ values of 2.9 and 6.4 μM, respectively [159].

Tannins possess not only liver and kidney protection properties, but also anti-diarrhea, anticancer, antibacterial and hypoglycemic activities [133]. However, a condensed tannin terminalin (**186**) from *T. oblongata* was reported to have severe hepatorenal toxicity and even caused renal necrosis [39].

### Flavonoids

**Fig. 3:**
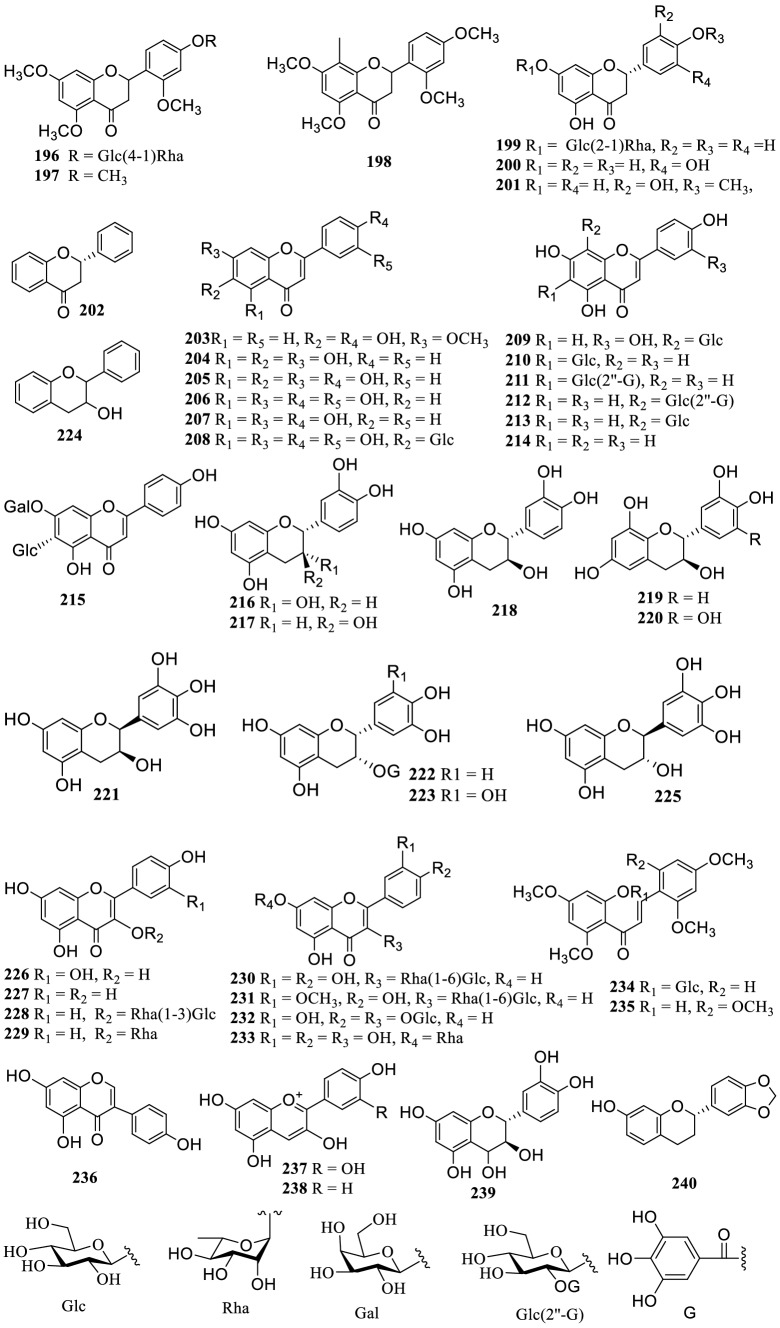
The structures of flavonoids **197–240**

The *Terminalia* genus are rich in flavonoids (Fig. 3) comprising of flavanones (**196–202**), flavones (**203–215**), flavan-3-ols (**216–225**), and flavonols (**226–233**). Among them, cerasidin (**235**) of chalcone, genistein (**236**) of isoflavone, and leucocyanidin (**239**) of flavan-3,4-diol from *T. arjuna* [80] were described as rare structural types in the *Terminalia* genus. Moreover, a new chalcone glycoside 2-*O*-β-glucosyloxy-4,6,2′,4′-tetramethoxychalchone (**234**) was reported from the roots of *T. alata* [53]. In addition, anthocyanidin cyanidin (**237**) and pelargonidin (**238**), flavanoid 7-hydroxy-3′,4-(methylenedioxy)flavan (**240**) and other structure were reported [12, 23, 66]. Compounds **209–213**, **215** were *C*-glycosides at C-6 or C-8 of ring A.

### Lignans

**Fig. 4:**
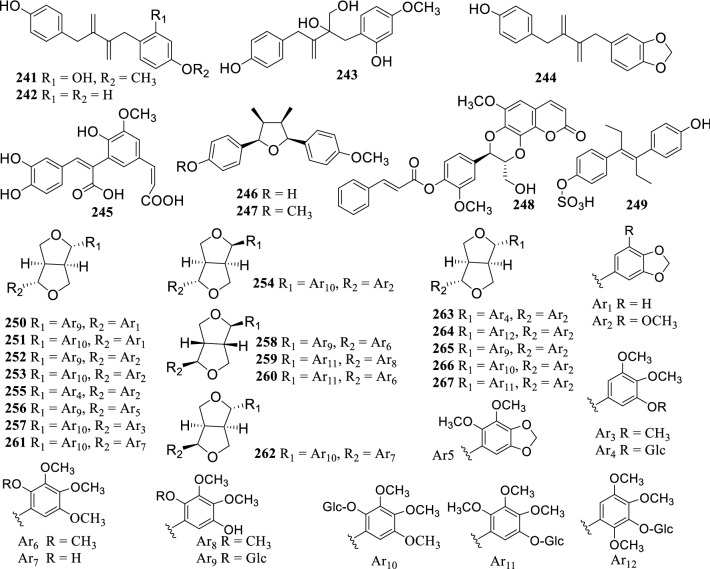
The structures of lignans **241–267**

Twenty-seven lignans (**241–267**) were reported from the genus *Terminalia* (Fig. 4). A new lignan 4′-*O*-cinnamoyl cleomiscosin A (**248**) was reported from the ethanol extract of *T. tropophylla* roots [72]. Moreover, 13 new furofuran lignan glucosides, terminalosides A–K (**250–260**), 2-epiterminaloside D (**261**), 6-epiterminaloside K (**262**) and 5 new polyalkoxylated furofuranone lignan glucosides, terminalosides L–P (**263–267**) were obtained from the leaves of *T. citrina*. All of them were tested for their estrogenic and/or antiestrogenic activities using estrogen responsive breast cancer cell lines T47D and MCF-7, and showed varying degrees of inhibitory activity. Among them, terminalosides B (**251**), G (**256**), L (**263**) and M (**264**) inhibited cell growth by up to 90% at a minimum concentration of 10 nM [22, 121].

### Phenols and Glycosides

**Fig. 5:**
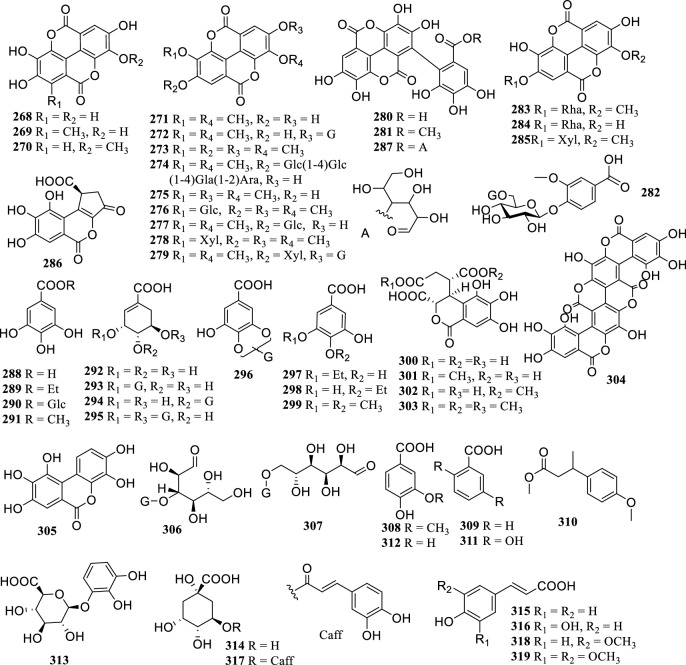
The structures of phenols and glycosides (**268–319**)

There are 52 phenols and glycosides reported in the *Terminalia* genus (Fig. 5), in which ellagic acid (**268**) and gallic acid (**289**) are present in almost all species. Studies have shown that most of the simple phenolic compounds have antioxidant, antibacterial, hypoglycemic, liver and kidney protection [23].

### Sterols and Cardiac Glycosides

**Fig. 6:**
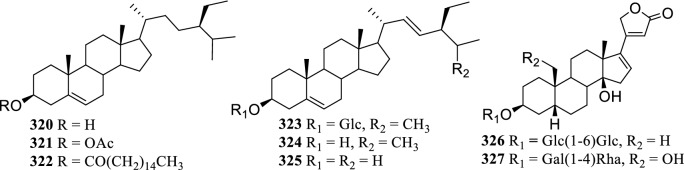
The structures of steroids (**320–325**) and cardiac glycosides (**326–327**)

Only 6 sterols (**320–325**) and 2 cardiac glycosides (**326-327**) were isolated from the genus *Terminalia* before 2001 (Fig. 6).

### Polyols and Esters

**Fig. 7:**
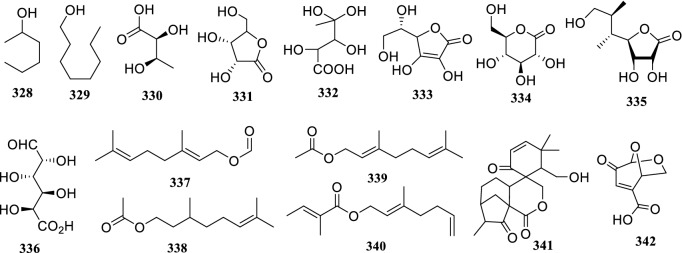
The structures of polyols and esters (**328–342**)

Polyols and lipids were reported to be abundant in the genus *Terminalia* and concentrated mainly in fruits and leaves [125]. So far, 9 polyol (**328–336**) and 6 esters (**337–342**) have been documented (Fig. 7).

### Other Compounds

**Fig. 8:**
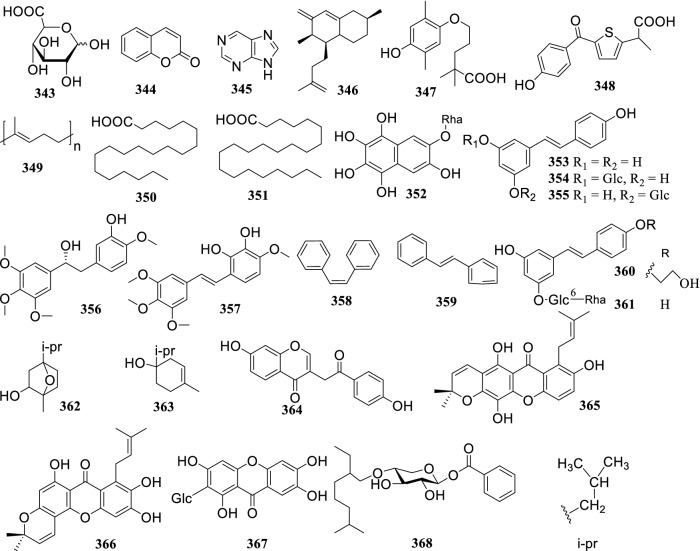
The structures of other compounds (**343–368**)

Other compounds featured in the *Terminalia* genus are shown in Fig. 8 and are mostly styrenes. Cao et al. isolated two new cytotoxic xanthones - termicalcicolanone A (**365**), termicalcicolanone B (**366**) in *T. calcicola*, and found an inhibitory effect on ovarian cancer [19]. Hiroko Negishi et al. obtained a new chromone derivative - terminalianone (**364**) from the barks of *Terminalia brownii* [98]. Ansari et al. isolated the novel compound, 4′-substituted benzoyl-β-d glycoside (**368**), from the fruits of *T. bellirica* and illustrated its potential for anticoagulation [160].

Moreover, chlorophyll and various vitamins were reported from the genus *Terminalia*.

## Pharmacological Activities

The pharmacological activities of the genus *Terminalia*, mainly including antimicrobial, antioxidant, cytotoxicity, anti-inflammatory, hypoglycemic, cardiovascular, mosquitocidal and antiviral, have been extensively studied.

### Antimicrobial

Extracts of several *Terminalia* species exhibit antimicrobial activity against various microbes. For example, methanol and aqueous extracts of *T. australis* were demonstrated antimicrobial activity against *Ca. albicans* (MIC = 180 and 250 µg/mL, resp.) and *Ca. kruzzei* (MIC = 250 and 300 µg/mL, resp.) [8]. Aqueous extracts of the stem barks, woods and whole roots of *T. brownii* showed antibacterial activity against standard strains of *Sta. aureus* (14.0 ± 1.1 µg/mL), *Escherichia coli*, *Ps. aeruginosa* (12.0 ± 1.1 µg/mL), *Klebsiella pneumonia* (6.0 ± 1.0 µg/mL), *Sa. typhi* and *Bacillus anthracis* (13.0 ± 1.0 µg/mL), as well as fungi *Ca. albicans* (12.3 ± 1.5 µg/mL) and *Cr. neoformans* (9.7 ± 1.1 µg/mL) [16]. Ethanol extracts of the root barks and leaves of *T. schimperiana* were against *Sta. aureus*, *Ps. aeruginosa* and *Sa. typhi* (MIC = 0.058–2.089 mg/mL), with inhibition zone diameters (IZDs) of 17.2 to 10.0 mm, compared to gentamicin (IZD = 21.8–10 mm). The results supported the efficacy of the extracts in the folkloric treatment of burns wounds, bronchitis and dysentery, respectively [42]. Antibacterial tests on *Mycobacterium smegmatis* ATCC 14468 showed that methanol extract of *T. sambesiaca* roots and stem barks had promising effects (MIC = 1.25 mg/mL, both) [133].

Ellagitannin punicalagin (**133**) obtained from the stem barks of *T. mollis* demonstrated crucial activity against *Ca. parapsilosis* and *Ca. krusei* (MIC = 6.25 μg/mL), as well as *Ca. albicans* (MIC = 12.5 μg/mL) [35]. 7-Hydroxy-3′,4′-(methylenedioxy) flavan (**240**), termilignan (**241**), anolignan B (**242**) and thannilignan (**243**) isolated from the fruit rinds of *T. bellirica* displayed significant antifungal activity against *Penicillium expansum* (MIC = 1.0, 2.0, 3.0 and 4.0 µg/mL, resp.), also with **240** and **241** against *Ca. albicans* at 10 and 6 µg/mL, resp. [12]. The antimycobacterial activity of friedelin (**79**) furnished from the root barks of *T. avicennioides* was 4.9 μg/mL in terms of MIC value [93]. *β*-Arjungenin (**16**), betulinic acid (**74**), sitosterol (**319**) and stigmasterol (**323**) from *T. brownii* were proved to possess antibacterial activity, with **74** the most active against *A. niger* and *S. ipomoea* (MIC = 50 μg/ml) [99].

### Antioxidant

*Terminalia* species have also illustrated some interesting antioxidant properties [161]. By a 2,2-diphenyl-1-picrylhydrazyl (DPPH) radical scavenging assay, relatively high anti-oxidant activities of the methanol extracts of *T. alata*, *T. bellirica* and *T. corticosa* trunk-barks were found (IC_50_ = 0.24, 1.02 and 0.25 mg/mL, resp.), compared to the positive control, l-ascorbic acid (IC_50_ = 0.24 mg/mL) [2].

Flavonoid glycosides, apigenin-6-*C*- (**211**) and apigenin-8-*C*- (**212**) (2″-*O*-galloy1)-*β*-d-glucoside, isolated from dried fallen leaves of *T. catappa*, showed significant antioxidative effects (IC_50_ = 2.1 and 4.5 µM, resp.) on Cu^2+^/0^2^-induced low density lipoprotein lipid peroxidation, with probucol (IC_50_ = 4.0 µM) as positive control [105].

Arjunaphthanoloside (**351**), isolated from the stem barks of *T. arjuna* showed potent antioxidant activity and inhibited nitric oxide (NO) production in lipopolysaccharide (LPS)-stimulated rat peritoneal macrophages [87], while ivorenosides B (**51**) and C (**52**), two triterpenoid saponins from *T. ivorensis*, exhibited scavenging activities against DPPH and ABTS^+^ radicals [131].

The antioxidant potential of *T. paniculata* (TPW) was investigated by DPPH, ABTS^2−^, NO, superoxide (O^2−^), Fe^2+^ chelating and ferric reducing/antioxidant power (FRAP) assays. TPW showed maximum superoxide, ABTS^2−^, NO, DPPH inhibition, and Fe^2+^-chelating property at 400 µg/mL, resp. FRAP value was 4.5 ± 0.25 µg Fe(II)/g, which demonstrated the efficacy of aqueous barks extract of *T. paniculata* as a potential antioxidant and analgesic agent [142].

TaB contains various natural antioxidants and has been used to protect animal cells against oxidative stress. The alleviating effect of TaB aqueous extract against Ni toxicity in rice (*Oryza sativa* L.) suggested that TaB extract considerably alleviated Ni toxicity in rice seedlings by preventing Ni uptake and reducing oxidative stress in the seedlings [162]. Behavioral paradigms and PCR studies of TaB extract against picrotoxin-induced anxiety showed that TaB supplementation increased locomotion towards open arm (EPM), illuminated area (light–dark box test), and increased rearing frequency (open field test) in a dose dependent manner, compared to picrotoxin (P < 0.05). Furthermore, alcoholic extract of TaB showed protective activity against picrotoxin in mice by modulation of genes related to synaptic plasticity, neurotransmitters, and antioxidant enzymes [174].

### Cytotoxicity

70% Acetone extracts of *T. calamansanai* leaves inhibited the viability of human promyelocytic leukemia HL-60 cells. Sanguiin H-4 (**115**), 1-*α*-*O*-galloylpunicalagin (**136**), punicalagin (**135**), 2-*O*-galloylpunicalin (**147**) and methyl gallate (**290**) were the main components isolated from *T. calamansanai* with the IC_50_ values of 65.2, 74.8, 42.2, 38.0 and > 100 µM, respectively, for HL-60 cells. Apoptosis of HL-60 cells treated with 1-*α*-*O*-galloylpunicalagin, **115**, **135**, and **147** was noted by the appearance of a sub-G1 peak in flow cytometric analysis and DNA fragmentation by gel electrophoresis. **115** and **147** induced a decrease of the human poly (ADP-ribose) polymerase (PARP) cleavage-related procaspase-3 and elevated activity of caspase-3 in HL-60 cells, but not normal human peripheral blood mononuclear cells, PBMCs [18].

Terminaliaside A (**60**), an oleanane-type triterpenoid saponin isolated from the roots of *T. tropophylla* showed antiproliferative activity against the A2780 human ovarian cancer cell line with an IC_50_ value of 1.2 µM [72]. The 70% methanolic extract of *T. chebula* fruits was found to decrease cell viability, inhibit cell proliferation, and induce cell death of human (MCF-7) and mouse (S115) breast cancer, human osteosarcoma (HOS-1), human prostate cancer (PC-3) and a non-tumorigenic, immortalized human prostate (PNT1A) cell lines. Flow cytometry and other analyses showed that some apoptosis was induced by the extract at lower concentrations, but at higher concentrations, necrosis was the major mechanism of cell death. Chebulinic acid (**143**) and ellagic acid (**186**) were tested by ATP assay on HOS-1 cell line in comparison with three known antigrowth phenolics of *Terminalia*, gallic acid (**287**), methyl gallate (**290**), luteolin (**206**), and tannic acid (**169**). Results showed that the most growth inhibitory phenolics in *T. chebula* fruits were chebulinic acid (IC_50_ = 53.2 µM ±/0.16) >/tannic acid (IC_50_ = 59.0 mg/mL ±/0.19) > ellagic acid (IC_50_ = 78.5 µM ±/0.24) [111].

Aqueous and ethanolic extracts of *T. citrina* fruits were revealed to exhibit significant mutagenicity in tested strains of baby hamster kidney cell line (BHK-21). Ethanolic extract showed higher mutagenicity in TA 100 strain, whereas aqueous extract exhibited higher mutagenicity in TA 102 strain than TA 100. Both extracts showed dose-dependent mutagenicity. Fifty percent cell viability was exhibited by 260 and 545 μg/mL of ethanolic and aqueous extracts respectively [169]. Moreover, ivorenoside A (**50**) showed antiproliferative activity against MDA-MB-231 and HCT116 human cancer cell lines with IC_50_ values of 3.96 and 3.43 µM, respectively [131].

### Anti-inflammatory

Inflammation has been considered as a major risk factor for various kinds of human diseases. Macrophages play substantial roles in host defense against infection. It can be activated by LPS, the major component of the outer membrane of Gram-negative bacteria. An investigation was carried out to determine anti-inflammatory potential of ethyl acetate fraction isolated from *T. bellirica* (EFTB) in LPS stimulated RAW 264.7 macrophage cell lines. EFTB (100 μg/mL) inhibited all inflammatory markers in dose dependent manner. Moreover, EFTB down regulated the mRNA expression of TNF-α, IL-6, COX-2 and NF-κB against LPS stimulation. These results demonstrated that EFTB is able to attenuate inflammatory response possibly via suppression of ROS and NO species, inhibiting the production of arachidonic acid metabolites, proinflammatory mediators and cytokines release [165].

Anolignan B (**242**) isolated from roots of *T. sericea* was tested for anti-inflammatory activity using the cyclooxygenase enzyme assays (COX-1 and COX-2) It showed activity against both COX-1 (IC_50_ = 1.5 mM) and COX-2 (IC_50_ = 7.5 mM) enzymes [151]. Termiarjunosides I (**47**) and II (**48**) isolated from stem barks of *T. arjuna* inhibited aggregation of platelets and suppressed the release of NO and superoxide from macrophages [156].

The anti-inflammatory activities of a polyphenol-rich fraction (TMEF) obtained from *T. muelleri* was assessed using carrageenan-induced paw edema model by measuring PGE2, TNF-α, IL-1b, and IL-6 plasma levels as well as the paw thickness. The group treated with 400 mg/kg of TMEF showed a greater inhibition in the number of writhes (by 63%) than the standard treated group (61%). TMEF pretreatment reduced the edema thickness by 48, 53, and 62% at the tested doses, respectively. TMEF administration inhibited the carrageenan-induced elevations in PGE2 (by 34, 43, and 47%), TNF-α (18, 28, and 41%), IL-1β (14, 22, and 29%), and IL-6 (26, 31, and 46%) [166].

### Hypoglycemic

Some species and isolates from *Terminalia* have indicated possession of α-glucosidase inhibitory capabilities. Gallic acid (**287**) and methyl gallate (**290**), from stem barks of *T. superba*, showed significant activity (IC_50_ = 5.2 ± 0.2 and 11.5 ± 0.1 μM, resp.). Arjunic acid (**5**) and glaucinoic acid (**46**) from stem barks of *T. glaucescens* showed significant *β*-glucuronidase inhibitory activity with IC_50_ value 80.1 and 500 μM, resp., against *β*-glucuronidase [130].

In a study to investigate *α*-glucosidase inhibition of extracts and isolated compounds from *T. macroptera* leaves, chebulagic acid (**142**) showed an IC_50_ value of 0.05 µM towards *α*-glucosidase and 24.9 ± 0.4 µM towards 15-lipoxygenase (15-LO), in contrast to positive controls (acarbose: IC_50_ = 201 ± 28 µM towards *α*-glucosidase, quercetin: IC_50_ = 93 ± 3 µM towards 15-LO). Corilagin (**116**) and narcissin (**231**) were good 15-LO and *α*-glucosidase inhibitors. Rutin (**230**) was a good *α*-glucosidase inhibitor (IC_50_ ca. 3 µM), but less active towards 15-LO [136].

From the fruits of *T. chebula*, 23-*O*-galloylarjunolic acid (**30**) and 23-*O*-galloylarjunolic acid 28-*O*-*β*-d-glucosyl ester (**31**) were afforded and showed potent inhibitory activities with IC_50_ values of 21.7 (**30**) and 64.2 (**31**) µM, resp., against Baker’s yeast *α*-glucosidase, compared to the positive control, acarbose (IC_50_ 174.0 µM) [146].

Hydrolyzable tannins, 1,2,3,6-tetra-*O*-galloyl-4-*O*-cinnamoyl-*β*-d-glucose (**183**) and 4-*O*-(2″,4″-di-*O*-galloyl-α-l-rhamnosyl) ellagic acid (**186**) from the fruits of *T. chebula*, showed significant *α*-glucosidase inhibitory activities with IC_50_ values of 2.9 and 6.4 µM, resp. In addition, inhibition kinetic studies showed that both compounds have mixed-type inhibitory activities with the inhibition constants (Ki) of 1.9 and 4.0 µM, respectively [159].

### Cardiovascular

A few species of *Terminalia* have demonstrated cardiovascular activities. It was reported that the barks of *T. arjuna* possessed significant inotropic and hypotensive effect, mild diuretic, antithrombotic, prostaglandin E2 enhancing and hypolipidaemic activities [66].

Ethanolic extract of *T. pallida* fruits (TpFE) were studied to determine their cardioprotection against isoproterenol (ISO)-administered rats. The supplementation of TpFE dose-dependently exerts notable protection on myocardium by virtue of its strong antioxidant activity. It could be used as a medicinal food for the treatment of cardiovascular ailments [163].

### Mosquitocidal

Insect-borne diseases remain to this day a major source of illness and can cause death worldwide. The resistance to chemical insecticides among mosquito species has been a major problem in vector control. The larvicidal and ovicidal activities of crude benzene, hexane, ethyl acetate, chloroform and methanol extracts of *T. chebula* were tested for their toxicity against three important vector mosquitoes, viz., *Anopheles stephensi*, *Aedes aegypti* and *Culex quinquefasciatus*. All extracts showed moderate larvicidal effects, the highest larval mortality was found in the methanol extract of *T. chebula* against the larvae of *A. stephensi*, *A. aegypti*, and *C. quinquefasciatus* with the LC_50_ values of 87.13, 93.24 and 111.98 ppm, respectively. Mean percent hatchability of the ovicidal activity was observed 48 h post treatment. All the five solvent extracts showed moderate ovicidal activity. The maximum egg mortality (zero hatchability) was observed in the methanol extract of *T. chebula* at 200 and 250 ppm against *A. stephensi*, while *A. aegypti* and *C. quinquefasciatus* showed 100% mortality at 300 ppm. No mortality was observed in the control group. The finding of the investigation revealed that the leaf extract of *T. chebula* possesses remarkable larvicidal and ovicidal activity against medically important vector mosquitoes [167, 168].

### Antiviral

Termilignan (**241**) and anolignan B (**242**), obtained from *T. bellirica* exhibited antimalarial activity against the chloroquine-susceptible strain 3D7 of *Plasmodium falciparum* (IC_50_ = 9.6 ± 1.2 μM)[12]. Casuarinin (**129**), chebulagic acid (**142**) from the fruits of *T. chebula* possessed hepatitis C virus inhibition activities (IC_50_ = 9.6 and 5.2 μM, resp.) [118]. Punicalin (**128**) and 2-*O*-galloylpunicalin (**147**), isolated from aqueous extract of *T. triflora* leaves, showed inhibitory activity on HIV-1 reverse transcriptase with IC_50_ of 0.11 μg/mL (0.14 μM) and 0.10 μg/mL (0.11 μM), resp. [149].

In vitro anti-HIV-1 activity of acetone and methanol extracts of *T. paniculata* fruits was studied by Durge A. et al. Cytotoxicity tests were conducted on TZM-bl cells and PBMCs, the CC_50_ values of both extracts were ≥ 260 μg/mL. By using TZM-bl cells, the extracts were tested for their ability to inhibit replication of two primary isolates HIV-1 (X4, Subtype D) and HIV-1 (R5, Subtype C). The activity against HIV-1 primary isolate (R5, Subtype C) was confirmed by using activated PBMC and quantification of HIV-1 p24 antigen. Both the extracts showed anti-HIV-1 activity in a dose-dependent manner. The EC_50_ values of the acetone and methanol extracts of *T. paniculata* were ≤ 10.3 μg/mL. Furthermore, the enzymatic assays were performed to determine the mechanism of action which indicated that the anti-HIV-1 activity might be due to inhibition of reverse transcriptase (≥ 77.7% inhibition) and protease (≥ 69.9% inhibition) enzymes [172].

Kesharwani A. et al. investigated anti-HSV-2 activity of *T. chebula* extract and its constituents, chebulagic acid (**142**) and chebulinic acid (**143**). Cytotoxicity assay using Vero cells revealed CC_50_ = 409.71 ± 47.70 μg/mL for the extract whereas **142** and **143** showed more than 95% cell viability up to 200 μg/mL. The extract from *T. chebula* (IC_50_ = 0.01 ± 0.0002 μg/mL), chebulagic (IC_50_ = 1.41 ± 0.51 μg/mL) and chebulinic acids (IC_50_ = 0.06 ± 0.002 μg/mL) showed dose dependent in vitro anti-viral activity against HSV-2, which can also effectively prevent the attachment and penetration of the HSV-2 to Vero cells. In comparison, acyclovir showed poor direct anti-viral activity and failed to significantly (p > 0.05) prevent the attachment as well as penetration of HSV-2 to Vero cells when tested up to 50 μg/mL. Besides, in post-infection plaque reduction assay, *T. chebula* extract, chebulagic and chebulinic acids showed IC_50_ values of 50.06 ± 6.12, 31.84 ± 2.64, and 8.69 ± 2.09 μg/mL, resp., which were much lower than acyclovir (71.80 ± 19.95 μg/mL) [173].

### Others

*Terminalia* species were also reported to be used in the treatment of diarrhea [95], Alzheimer’s disease [112], psoriasis [164], liver disease [170], kidney disease [171], etc. Terminalosides A–K (**249–259**) from the leaves of the Bangladeshi medicinal plant *T. citrina* possess estrogen-inhibitory properties. Among them, Terminaloside E (**253**) showed inhibitory activity against the T47D cell line, such terminalosides C (**252**), F (**255**), and I (**258**). Besides, 6-epiterminaloside K (**262**) displayed antiestrogenic activity against MCF-7 cells [22].

## Conclusion and Future Prospects

The genus *Terminalia* contains not only a large number of tannins, simple phenolics, but also a lot of terpenoids, flavonoids, lignans and other compounds. Most tannins, simple phenolics and flavonoids have antioxidation, antibacterial, antiinflammatory and anticancer activities. The plants of the genus *Terminalia* have exhibited positive effect on immune regulation, cardiovascular disease and diabetes, and can accelerate wound healing [157]. Therefore, the *Terminalia* genus has great medicinal potential. However, most of the chemical composition of species is still unknown, we should use modern advanced technology such as LC–MS to continue to isolate its compounds, and determine their pharmacological activities and mechanism of action, to explore other possible greater medicinal value.

## Abbreviations

*A.*: *Aspergillus*
BCG: *Bacillus Calmette Guerin*
BMM: Broth microdilution method
*Ca.*: *Candida*
*Cr.*: *Cryptococcus*
CC_50_: Cytotoxic concentration of the extracts to cause death to 50% of host’s viable cells
DPPH: 2,2-Diphenyl-1-picrylhydrazyl
*E.*: *Escherichia*
EC_50_: Half maximal effective concentration
FRAP: Ferric reducing/antioxidant power
GABA: Neurotransmitter gamma-aminobutyric acid
IC_50_: Minimum inhibition concentration for inhibiting 50% of the pathogen
*K.*: *Klebsiella*
MIC: Minimum inhibitory concentration
MTT: 3-(4,5-Dimethylthiazol-2-yl)-2,5-diphenyl tetrazolium bromide
*Ps.*: *Pseudomonas*
*Sa.*: *Salmonella*
*Sta.*: *Staphylococcus*
*Str.*: *Streptomyces*

## References

[1] McGaw LJ, Rabe T, Sparg SG, Jäger AK, Eloff JN, van Staden J (2001). J. Ethnopharmacol..

[2] Nguyen Q, Nguyen VB, Eun J, Wang S, Nguyen DH, Tran TN, Ngugen AD (2016). Res. Chem. Intermed..

[3] Srivastava SK, Chouksey BK, Srivastava SD (2001). Fitoterapia.

[4] Arias D, Calvo-Alvarado J, de Richter DB, Dohrenbusch A (2011). Biomass Bioenergy.

[5] Y. Noboru, I. Ayako, K. Chika, S. Keisuke, JP2001098264 A 20010410 (2001)

[6] Yadav RN, Rathore K (2001). Phytochem. Commun..

[7] S. Fangwen, P. Raoji, CN105232959 A 20160113 (2016)

[8] Carpano SM, Spegazzini ED, Rossi JS, Castro MT, Debenedetti SL (2003). Fitoterapia.

[9] Omonkhua AA, Cyril-Olutayo MC, Akanbi OM, Adebayo OA (2013). Parasitol. Res..

[10] Zanna H, Ahmad S, Abdulmalik B (2014). Tasi’u M, Abel GO, Musa HM. Adv. Biochem..

[11] Chopra RN, Nayar SL, Chopra IC (1956). Glossary of Indian Medicinal Plants.

[12] Valsaraj R, Pushpangadan P, Smitt UW, Adsersen A, Christensen SB, Sittie A, Nyman U, Nielsen C, Olsen CE (1997). J. Nat. Prod..

[13] Gurib-Fakim A (1994). J. Essent. Oil Res..

[14] Khatoon S, Singh N, Srivastava N, Rawat A, Mehrotra S (2008). J. Planar Chromatogr..

[15] Gelfand M (1985). The Traditional Medical Practitioner in Zimbabwe, His Principles of Practice and Pharmacopoeia.

[16] Mbwambo ZH, Moshi MJ, Masimba PJ, Kapingu MC, Nondo RS (2007). BMC Complement. Altern. Med..

[17] McIntosh KS (1934). Qld Agric. J..

[18] Chen LG, Huang WT, Lee LT, Wang CC (2009). Toxicol. Vitro.

[19] Cao S, Brodie PJ, Miller JS, Randrianaivo R, Ratovoson F, Birkinshaw C, Andriantsiferana R, Rasamison VE, Kingston DGI (2007). J. Nat. Prod..

[20] Lin Y, Kuo Y, Shiao M, Chen C, Ou J (2000). J. Chin. Chem. Soc..

[21] Bag A, Bhattacharyya SK, Chattopadhyay RR (2013). Asian Pac. J. Trop. Biomed..

[22] Muhit MA, Umehara K, Moriyasumoto K, Noguchi H (2016). J. Nat. Prod..

[23] Singh A, Bajpai V, Kumar S, Kumar B, Srivastava M, Rameshkumar KB (2016). Ind. Crop Prod..

[24] Zhang T, Sun H (2011). J. Plant. Res..

[25] Konczak I, Maillot F, Dalar A (2014). Food Chem..

[26] Koudou J, Roblot G, Wylde R (1995). Planta Med..

[27] Okpekon T, Yolou S, Gleye C, Roblot F, Loiseau P, Bories C, Grellier P, Frappier F, Laurens A, Hocquemiller R (2004). J. Ethnopharmcol..

[28] O. Nobuhiko, S. Shizu, C. Nuan, JP2007099733 A 20070419 (2007)

[29] Adiko VA, Attioua BK, Tonzibo FZ, Assi KM, Siomenan C, Djakouré LA (2013). Afr. J. Biotechnol..

[30] J. Hutchinson, J.M. Dalziel, *Appendix to the Flora of West Tropical Africa* (Published on behalf of the Governments of Nigeria, Ghana, Sierra Leone and the Gambia by the Crown Agents for Oversea Governments and Administrations, London, 1936), p. 81

[31] Anam K, Widharna RM, Kusrini D (2009). Int. J. Pharmacol..

[32] Musa MS, Abdelrasool FE, Elsheikh EA, Ahmed LA, Mahmoud ALE, Yagi SM (2011). J. Med. Plants Res..

[33] Ogbazghi W, Bein E (2006). Drylands Coord. Group Rep..

[34] Zou Y, Ho GTT, Malterud KE, Le NHT, Inngjerdingen KT, Barsett H, Diallo D, Michaelsen TE, Paulsen BS (2014). J. Ethnopharmacol..

[35] Liu M, Katerere DR, Gray AI, Seidel V (2009). Fitoterapia.

[36] Anam K, Suganda AG, Sukandar EY, Kardono LBS (2010). Res. J. Med. Plant.

[37] Bajpai M, Pande A, Tewari SK, Prakash D (2005). Int. J. Food Sci. Nutr..

[38] Marzouk MSA, El-Toumy SAA, Moharram FA (2002). Planta Med..

[39] Oelrichs PB, Pearce CM, Zhu J, Filippich LJ (1994). Nat. Toxins.

[40] Malekzadeh F, Ehsanifar H, Shahamat M (2001). Int. J. Antimicrob. Agents.

[41] Lin TC, Hsu FL (1999). J. Chin. Chem. Soc..

[42] Nmema EE, Anaele EN (2013). J. Appl. Sci. Environ. Manag..

[43] Mongalo NI, McGaw LJ, Segapelo TV, Finnie JF, Van Staden J (2016). J. Ethnopharmacol..

[44] Fyhrquist P, Mwasumbi L, Haeggstrom CA, Vuorela H, Hiltunen R, Vuorela P (2002). J. Ethnopharmacol..

[45] Chhabra SC, Mahunnah RLA, Mshiu EN (1989). J. Ethnopharmacol..

[46] B. Heine, M. Brenzinger, *Plants of the Borana (Ethiopia and Kenya), Plant Concepts and Plant Use: An Ethno*-*botanical Survey of the Semi*-*arid and Arid Lands of East Africa: Part 4* (Verlag Brentenbach, 1988), p. 23

[47] Ndamba J, Lemmich E, Mølgaard P (1994). Phytochemistry.

[48] Katerere DR, Gray AI, Nash RJ, Waigh RD (2003). Phytochemistry.

[49] Tabopda TK, Ngoupayo J, Tanoli SAK, Mitaine-Offer AC, Ngadjui BT, Ali MS, Luu B, Lacaille-Dubois MA (2009). Planta Med..

[50] Wansi JD, Lallemand MC, Chiozem DD, Toze FAA, Mbaze LMA, Naharkhan S, Iqbal MC, Fomum ZT (2007). Phytochemistry.

[51] Jono T (2015). Curr. Herpetol..

[52] Srivastava SK, Srivastava SD, Chouksey BK (2001). Fitoterapia.

[53] Srivastava SK, Srivastava SD, Chouksey BK (1999). Fitoterapia.

[54] Anjaneyulu ASR, Reddy AVR, Mallavarapu RG, Chandrasekhara RS (1986). Phytochemistry.

[55] Mallavarapu GR, Rao SB, Syamasundar KV (1986). J. Nat. Prod..

[56] Mallavarapu GR, Muralikrishna E (1983). J. Nat. Prod..

[57] Anjaneyulu ASR, Prasad AVR (1983). Phytochemistry.

[58] Ali A, Kaur G, Hamid H, Abdullah T, Ali M, Niwa M, Alam MS (2003). J. Asian Nat. Prod. Res..

[59] Yadav RN, Rathore K (2001). Fitoterapia.

[60] Honda T, Murae T, Tsuyuki T (1976). Chem. Pharm. Bull..

[61] Honda T, Murae T, Tsuyuki T, Takahashi T, Sawai M (1976). Bull. Chem. Soc. Jpn.

[62] Anjaneyulu ASR, Prasad ASR (1982). J. Chem..

[63] Anjaneyulu ASR, Prasad AVR (1982). Phytochemistry.

[64] Sharma PN, Shoeb PN, Kapil RS (1982). Indian J. Chem..

[65] Pettit GR, Hoard MS, Doubek DL, Schmidt JM, Pettit RK, Tackett LP, Chapuis JC (1996). J. Ethnopharmacol..

[66] Dwivedi S (2007). J. Ethnopharmacol..

[67] Row LR, Murty PS, Rao GS, Sastry CP, Rao KVJ (1970). Indian J. Chem..

[68] Wang W, Ali Z, Li XC, Shen Y, Khan IA (2010). Planta Med..

[69] Aiyelaagbe O, Olaoluwa O, Oladosu I, Gibbons S (2014). Rec. Nat. Prod..

[70] Kalola J, Rajani M (2006). Chromatographia.

[71] Bombardelli E, Martinelli EM, Mustich G (1974). Phytochemistry.

[72] Cao S, Brodie PJ, Callmander M, Randrianaivo R, Rakotobe E, Rasamison VE, Kingston DG (2010). Phytochemistry.

[73] Nandy AK, Podder G, Sahu NP, Mahato SB (1989). Phytochemistry.

[74] Tsuyuki T, Hamada Y, Honda T, Takahashi T, Matsushita K (1979). Bull. Chem. Soc. Jpn.

[75] Jung SW, Shin MH, Jung JH, Kim ND, Im KS (2001). Arch. Pharmacal Res..

[76] Wang W, Ali Z, Shen Y, Li XC, Khan IA (2010). Fitoterapia.

[77] King FE, King TJ, Ross JM (1954). J. Chem. Soc..

[78] Row LR, Murty PS, Rao GSRS, Sastry CSP, Rao KVJ (1970). Indian J. Chem..

[79] Aggarwal RR, Dutt S (1936). Proc. Natl. Acad. Sci. U.S.A..

[80] Tripathi VK, Singh B, Tripathi RC, Upadhyay RK, Pandey VB (1996). Orient. J. Chem..

[81] Upadhyay RK, Pandey MB, Jha RN, Singh VP, Pandey VB (2001). J. Asian Nat. Prod. Res..

[82] Pawar RS, Bhutani KK (2005). Phytomedicine.

[83] Nagar A, Gujral VK, Gupta SR (1979). Planta Med..

[84] Ali A, Abdullah ST, Hamid H, Ali M, Alam MS (2003). Indian J. Chem..

[85] Chouksey BK, Srivastava SK (2001). Indian J. Chem..

[86] Kandil FE, Narsar NI (1998). Phytochemistry.

[87] Ali A, Kaur G, Hayat K, Ali M, Ather M (2003). Pharmazie.

[88] Link HY, Chang L (2005). Planta Med..

[89] Nair S, Nagar R (1997). South Asian J. Prev. Cardiol..

[90] Lin TC, Chien SC, Chen HF, Hsu FL (2000). Chin. Pharm. J..

[91] Mann A, Ibrahim K, Oyewale AO, Amupitan JO, Fatope MO, Okogun JI (2012). Am. J. Org. Chem..

[92] Shuaibu MN, Wuyep PT, Yanagi T, Hirayama K, Ichinose A, Tanaka T, Kouno I (2008). Parasitol. Res..

[93] Mann A, Ibrahim K, Oyewale AO, Amupitan JO, Fatope MO, Okogun JI (2011). Am. J. Chem..

[94] Yadava RN, Rathore K (2001). Fitoterapia.

[95] Pandey G, Gupta SS, Bhatia A, Sidhu OP, Rawat AKS, Rao CV (2017). J. Ethnopharmacol..

[96] Row JR, Murty PS (1970). Indian J. Chem..

[97] Elsayed HE, Akl MR, Ebrahim HY, Sallam AA, Haggag EG, Kamal AM, El Sayed KA (2015). Chem. Biol. Drug Des..

[98] Negishi H, Maoka T, Njelekela M, Yasui N, Juman S, Mtabaji J, Miki T, Nara Y, Yamori Y, Ikeda K (2011). J. Asian Nat. Prod. Res..

[99] Opiyo SA, Manguro LOA, Owuor PO, Ochieng CO, Ateka EM, Lemmen P (2011). Nat. Prod. J..

[100] Yamauchi K, Mitsunaga T, Muddathir AM (2016). J. Wood Sci..

[101] Salih EYA, Kanninen M, Sipi M, Luukkanen O, Hiltunen R, Vuorela H, Julkunen-Tiitto R, Fyhrquist P (2017). S. Afr. J. Bot..

[102] Tanaka T, Morita A, Nonaka GI, Lin TC, Nishioka I, Ho FC (1991). Chem. Pharm. Bull..

[103] Chen LG, Wang CC, Yang LL (2004). Abstr. Pap. Am. Chem. Soc..

[104] Lin TC, Ma YT, Hsu FL (1996). Chin. Pharm. J. (Taipei).

[105] Chen PS, Li JH, Liu TY, Lin TC (2000). Cancer Lett..

[106] Chandrasekhar Y, Ramya EM, Navya K, Kumar GP, Anilakumar KR (2017). Biomed. Pharmacother..

[107] Tanaka T, Nonaka GI, Nishioka I (1986). Chem. Pharm. Bull..

[108] *Dukes Phytochemical and Ethnobotanical Database* (2008), p. 11

[109] Mandloi S, Mishra R, Varma R, Varughese B, Tripathi J (2013). Int. J. Pharm. Bio Sci..

[110] Han Q, Song J, Qiao C, Wong L, Xu H (2006). J. Sep. Sci..

[111] Saleem A, Husheem M, Härkönen P, Pihlaja K (2002). J. Ethnopharmacol..

[112] Afshari AR, Sadeghnia HR, Mollazadeh H (2016). Adv. Pharmacol. Sci..

[113] Baliga MS (2010). J. Altern. Complement. Med..

[114] Sornwatana T, Bangphoomi K, Roytrakul S, Wetprasit N, Choowongkomon K, Ratanapo S (2015). Biotechnol. Appl. Biochem..

[115] Mammen D, Bapat S, Sane R (2012). Int. J. Pharm. Bio Sci..

[116] Chandan S (1990). Phytochemistry.

[117] Khare CP (2004). Botany, Indian Herbal Remedies: Rational Western Therapy, Ayurvedic, and Other Traditional Usage.

[118] Ajala OS, Jukov A, Ma CM (2014). Fitoterapia.

[119] Juang LJ, Sheu SJ, Lin TC (2004). J. Sep. Sci..

[120] Burapadaja S, Bunchoo A (1995). Planta Med..

[121] Muhit MA, Umehara K, Noguchi H (2016). Fitoterapia.

[122] Konczak I, Zabaras D, Dunstan M, Aguas P (2010). Food Chem..

[123] Cunningham AB, Garnett S, Gorman J, Courtenay K, Boehme D (2009). Econ. Bot..

[124] Martino VS, Graziano MN, Hnatyszyn O, Coussio JD (1975). Planta Med..

[125] Rayan P, Matthews B, McDonnell PA, Cock IE (2015). Parasitol. Res..

[126] Sirdaarta J, Matthews B, Cock IE (2015). J. Funct. Food.

[127] Ekong DEU, Idemudia OG (1967). J. Chem. Soc. C.

[128] Idemudia OG (1970). Phytochemistry.

[129] Zareen S, Choudhary MI, Ngounou FN, Yasin A, Parvez M (2002). Tetrahedron Lett..

[130] Rahman AU, Zareen S, Choudhary MI, Akhtar MN, Ngounou FN (2005). Z. Nat. B.

[131] Ponou BK, Teponno RB, Ricciutelli M, Quassinti L, Bramucci M, Lupidi G, Barboni L, Tapondjou LA (2010). Phytochemistry.

[132] Ponou BK, Teponno RB, Ricciutelli M, Nguelefack TB, Quassinti L, Bramucci M, Lupidi G, Barboni L, Tapondjou LA (2011). Chem. Biodivers..

[133] Fyhrquist P, Laakso I, Marco SG, Julkunen-Tiitto R, Hiltunen R (2014). S. Afr. J. Bot..

[134] Muddathir AM, Yamauchi K, Mitsunaga T (2013). J. Wood Sci..

[135] Pham AT, Malterud KE, Paulsen BS, Diallo D, Wangensteen H (2011). Nat. Prod. Commun..

[136] Pham AT, Malterud KE, Paulsen BS, Diallo D, Wangensteen H (2014). Pharm. Biol..

[137] Conrad J, Vogler B, Reeb S, Klaiber I, Papajewski S, Roos G, Vasquez E, Setzer MC, Kraus W (2001). J. Nat. Prod..

[138] Conrad J, Vogler B, Klaiber I, Reeb S, Guse JH, Roos G, Kraus W (2001). Nat. Prod. Lett..

[139] Fahmy NM, Al-Sayed E, Abdel-Daim MM, Karonen M, Ingab AN (2016). Pharm. Biol..

[140] Majumdar K, Sinha MB, Som UK, Das S (2005). J. Indian Chem. Soc..

[141] Murdiati TB, McSweeney CS, Lowry JB (1992). Aust. J. Agric. Res..

[142] Talwar S, Nayak PG, Mudgal J, Paul P, Bansal P, Nandakumar K (2013). Nandakumar. J. Med. Food.

[143] Row LR, Raju RR (1967). Tetrahedron.

[144] Mopuri R, Ganjayi M, Banavathy KS, Parim BN, Meriga B (2015). BMC Complement. Altern. Med..

[145] Conrad J, Vogler B, Klaiber I, Roos G, Walter U, Kraus W (1998). Phytochemistry.

[146] Lee DY, Woo KH, Yang HJ, Hyun SS (2017). Bioorg. Med. Chem. Lett..

[147] Ramachandra RL, Ramkrishnam RR (1965). Curr. Sci..

[148] Row LR, Rao GS (1962). Tetrahedron.

[149] Martino V, Morales J, Martínez-Irujo JJ, Font M, Monge A, Coussio J (2004). Phytother. Res..

[150] Moshi MJ, Mbwambo ZH (2005). J. Ethnopharmacol..

[151] Eldeen IM, Elgorashi EE, Mulholland DA, van Staden J (2006). J. Ethnopharmacol..

[152] Joseph CC, Moshi MJ, Innocent E, Nkunya MHH (2007). Afr. J. Tradit. Complement. Altern. Med..

[153] Kuete V, Tabopda TK, Ngameni B, Nana F, Tshikalange TE, Ngadjui BT (2010). S. Afr. J. Bot..

[154] Li TC, Hsu FL (1996). Chin. Pharm. J..

[155] Wu LF, Yuan YB, Wang KF, Li SQ, Liu J, Zhang LZ, She GM (2014). Chin. Tradit. Herb. Drugs.

[156] Alam MS, Kaur G, Ali A, Hamid H, Ali M, Athar M (2008). Nat. Prod. Res..

[157] Cock IE (2015). Inflammopharmacology.

[158] Tchuenmogne MT, Kammalac T, Gohlke S, Kouipou R, Aslan A, Kuzu M, Sewald N (2017). Medicines (Basel).

[159] Lee DY, Kim HW, Yang H, Sung SH (2017). Phytochemistry.

[160] Ansari VA, Arif M, Hussain MS, Siddiqui HH, Dixit RK (2016). Integr. Med. Res..

[161] Rice-Evans C, Miller N, Paganga G (1997). Trends Plant Sci..

[162] Rajpoot R, Rani A, Srivastava RK, Pandey P, Dubey RS (2016). Protoplasma.

[163] Hussain SA, Kareem MA, Rasoo SN, Al Omar SY, Saleh A, Al-Fwuaires MA, Devi KL (2018). Biol. Trace Elem. Res..

[164] An J, Li T, Dong Y, Li Z, Huo J (2017). Cell. Physiol. Biochem..

[165] Jayesh K, Helen LR, Vysakh A, Binil E, Latha MS (2017). Biomed. Pharmacother..

[166] Fahmy NM, Al-Sayed E, Abdel-Daim MM, Singab AN (2017). Drug Dev. Res..

[167] Veni T, Pushpanathan T, Mohanraj J (2017). J. Parasit. Dis..

[168] Thanigaivel A, Vasantha-Srinivasan P, Senthil-Nathan S, Edwin ES, Ponsankar A, Chellappandian M, Kalaivani K (2017). Ecotoxicol. Environ. Saf..

[169] Akhtar MF, Saleem A, Sharif A, Akhtar B, Nasim MB, Peerzada S, Ali S (2016). EXCLI J..

[170] Kuriakose J, Raisa HL, Vysakh A, Eldhose B, Latha MS (2017). Biomed. Pharmacother..

[171] Mittal A, Tandon S, Singla SK, Tandon C (2017). Cytotechnology.

[172] Durge A, Jadaun P, Wadhwani A, Chinchansure AA, Said M, Thulasiram HV, Kulkarni SS (2017). Nat. Prod. Res..

[173] Kesharwani A, Polachira SK, Nair R, Agarwal A, Mishra NN, Gupta SK (2017). BMC Complement. Altern. Med..

[174] Sekhar YC, Kumar GP, Anilakumar KR (2017). Chin. J. Nat. Med..

